# A Review of Electrically Enhanced Oil Recovery (EEOR): Mechanism, Influencing Factors and Field Application Effects

**DOI:** 10.1021/acsomega.5c08342

**Published:** 2025-12-06

**Authors:** Dongyue Zhang, Qingjie Liu, Xinyu Zhou, Xiuxiu Pan, Jiale Shi

**Affiliations:** † 12381 University of Chinese Academy of Sciences, Beijing 100049, China; ‡ Institute of Porous Flow and Fluid Mechanics, Chinese Academy of Sciences, Langfang 065007, China; § Research Institute of Petroleum Exploration and Development, 205361 PetroChina, Beijing 100083, China

## Abstract

This paper presents a systematic review of electrically enhanced oil recovery (EEOR), focusing on the critical gap between its promising laboratory performance and the slow pace of industrial deployment. First, the current state of EEOR applications is reviewed. Although extensive laboratory research has confirmed its technical feasibility, field applications remain mostly confined to pilot-scale projects, particularly in heavy oil and oil sands reservoirs. These trials demonstrate EEOR’s potential to outperform conventional steam flooding in terms of recovery efficiency and energy utilization, but they also expose significant challenges—such as low reliability of downhole hardware (with failure rates reaching up to 75% in some projects), geomechanical instability, and operational difficulties—highlighting the technological gap between experimental success and large-scale reliability. Second, the review elucidates the fundamental mechanisms underlying EEOR. As a multiphysics coupled process, EEOR is governed by electrodynamic, electrothermal, and electrochemical effects. Key mechanisms include: electrodynamic effects (electroosmosis and electrophoresis), which provide nonpressure-driven flow forces; electrothermal effects (primarily Joule heating), which reduce heavy oil viscosity; and electrochemical effects (electrowetting and redox reactions), which alter wettability and enable in situ upgrading. A key insight is the inherent interplay—both antagonistic and synergistic—among these mechanisms, with reservoir water salinity serving as the central regulatory factor. The review further discusses emerging technologies, such as electromagnetic-assisted composite flooding and plasma pulse stimulation, emphasizing their potential for synergistic enhancement through multiphysics coupling. Third, economic and operational constraints are critically assessed. Although EEOR may offer energy cost advantages under specific conditions, its commercial viability is hindered by high electricity prices, substantial capital investment, and operational risks arising from equipment failures. Finally, this review outlines a forward-looking perspective. The advancement of EEOR depends on shifting the research focus from mechanism validation to resolving engineering bottlenecks. Future efforts should prioritize the development of highly reliable, long-lifetime downhole electrode and cable systems, the construction of quantitative models capable of predicting coupled multifield effects, and the integration of artificial intelligence for process optimization. Such an engineering-driven approach is essential for transforming EEOR from a promising laboratory concept into a practical, field-deployable technology.

## Graphical abstract

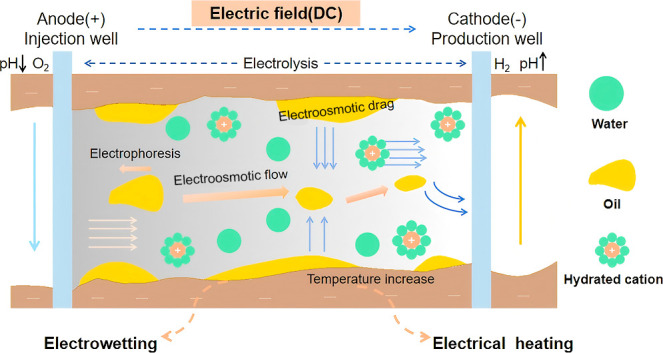

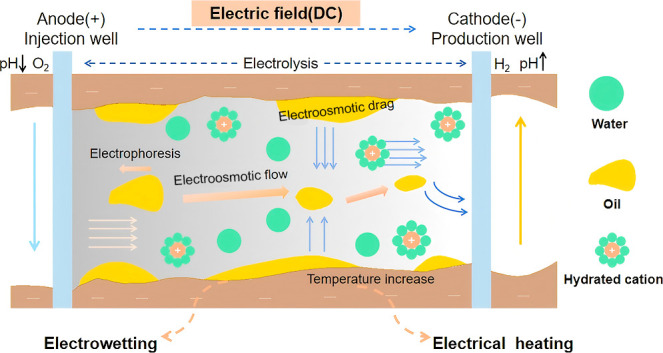

## 1 Introduction

Despite the ongoing transformation of the global energy structure, oil remains indispensable in the foreseeable future. However, after primary and secondary recovery, a substantial amount of residual oil remains trapped in the pore spaces, posing a significant challenge to further enhancing recovery rates. Consequently, there is a pressing need for advanced enhanced oil recovery (EOR) technologies.^1–3^ Currently, conventional EOR methods represented by thermal techniques (e.g., steam flooding), chemical methods (e.g., polymer/surfactant flooding), and gas methods (e.g., CO_2_ flooding) are the dominant technologies for global production enhancement.^4–7^ However, the dominance of these mainstream technologies does not equate to their universal applicability. Each technique faces distinct limitations, leaving a significant “technological gap” in many challenging reservoirs.

Thermal methods are highly effective for heavy oil but face severe heat loss issues in deep (>2500 feet) or thin reservoirs, alongside substantial consumption of water and energy, which also results in significant CO_2_ emissions.^8^ Chemical EOR technologies face significant constraints due to cost, reservoir adaptability, and technical risks. Core chemicals such as polymers and surfactants are relatively expensive, and their performance is highly sensitive to reservoir conditions. Polymers degrade and become ineffective under high salinity, high temperatures, and mechanical shear. Surfactants exhibit reduced stability in extreme environments. Alkali flooding readily reacts with reservoir constituents to form precipitates. Low-salinity water flooding is only suitable for highly clay-rich reservoirs, resulting in poor universality. Certain chemicals exhibit toxicity, and pore blockage may occur during injection due to adsorption or flocculation. Assessing chemical compatibility with reservoir formations further complicates technical implementation.^2,9,10^ Gas-driven EOR technology faces challenges in securing reliable gas sources and high costs. CO_2_ requires long-distance transportation, while the stability of nitrogen and hydrocarbon gas supplies impacts overall expenses. Strict reservoir compatibility is required, with CO_2_ demanding specific depths (2500–9800 feet) for miscibility.^11^ Nitrogen is prone to gravity separation, hydrocarbon gases to migration, and CO_2_ may corrode equipment. High initial capital investment and long payback periods result in poor economics during low oil price cycles, compounded by gas leakage risks and complex environmental assessment requirements.^2,10^ These limitations make it difficult to mobilize a significant amount of remaining oil using the current mainstream technologies. In this context, EEOR has emerged. It is not intended to completely replace traditional methods, but rather serves as an enabling technology with strategic potential, specifically targeting those reservoir types that are difficult to effectively develop with conventional methods. While we acknowledge that EEOR currently contributes only marginally to the global oil production, the need for in-depth research is crucial because its unique mechanisms hold the potential to solve the key pain points of traditional EOR, thus unlocking substantial “stranded” reserves. EEOR functions by applying an electric field inside the reservoir, inducing specific physicochemical effects in the target zone: electrodynamics effects provide nonpressure-driven forces in low-permeability media; electrical heating (such as Joule heating) enable efficient in situ heating, unconstrained by steam injection depth/thickness; and electrochemical processes (such as electrowetting and in situ modification) can alter the properties of fluids and rocks at the molecular level. This confers particular advantages in low-permeability and tight reservoirs and can be more economical and environmentally favorable by reducing chemical consumption and harmful byproducts.^12^ Note that “electrically enhanced oil recovery (EEOR)” and “electrokinetic EOR (EK-EOR)” are sometimes used interchangeably; strictly speaking, EK-EOR refers to recovery methods that act directly through electrokinetic effects, whereas EEOR is broader and can leverage electrical energy converted into other forms. In EK-EOR, the electrical current itself is the primary enhancement mechanism.^13^ The unique mechanism of EEOR offers clear application prospects for EOR technologies: 1.Targeting challenging reservoirs: EEOR offers new technical solutions for deep heavy oil, thin oil reservoirs, tight/low-permeability formations, and certain heterogeneous carbonate reservoirs, which are the weak points of traditional methods. 2. Environmental and efficiency advantages: as a potential “waterless” technology (compared to steam flooding), EEOR is expected to significantly reduce water consumption and related CO_2_ emissions. By directly generating heat within the reservoir, it also avoids the massive heat losses associated with deep-well steam injection, showing excellent energy efficiency in specific applications.^14^

Although the basic concept of EEOR dates back several decades, and there has been abundant laboratory research, its translation into large-scale field applications has been slow, mainly due to challenges such as instability of field effects, downhole tool reliability, and economic uncertainties. This gap between laboratory potential and field application highlights the need for critical analysis. Therefore, this review aims to (1) systematically summarize the research progress on the core mechanisms of EEOR (electrodynamics, electrical heating, electrochemistry) and their complex interactions; (2) assess the key factors affecting EEOR performance; (3) research and analyze the latest advancements and potential of advanced EEOR technologies (such as electromagnetic-assisted composite technology, plasma pulse technology, etc.); (4) conduct in-depth analysis of existing field application cases and perform economic evaluations, comparing theoretical predictions with actual performance to summarize successful experiences and ongoing challenges; (5) examine the prospects for hybrid EEOR methods and future technological directions. This review aims to provide a clear and exhaustive synopsis of EEOR. We delineate its prospective role, identify key developmental impediments, and propose a systematic blueprint for future research. The goal is to expedite EEOR’s transition from a laboratory concept to industrial reality.

## 2 Basic Mechanism of EEOR

### 2.1 Electrokinetic Phenomena

Electroosmosis flow (EOF) is one of the core drivers of EEOR. Since the rock surface is usually negatively charged, cations in the formation water gather near the rock wall to form a diffusion layer in the EDL, and the applied electric field drives the cations in the diffusion layer and their hydration layer to the cathode, driving the fluid in the entire pore space to macroscopic flow through viscous drag.^17^ EOF overcomes the reliance on differential pressure that characterizes conventional displacement methods. This makes it particularly effective in tight, low-permeability reservoirs, as it significantly reduces the required displacement pressure.^17^ In addition, for swelling clays, the electric field causes cations and water molecules—which maintain the clay’s swollen state—to move away from the clay minerals. This migration leads to dehydration, reduced swelling, and increased permeability.^15^ Ghazanfari et al. propose that EOF, driven by the EDL, generates momentum in the wetted phase (water), which is transferred to the nonwetted phase (oil) via viscous forces, a process termed viscous momentum transfer. The viscous force is the main driving force for transporting the oil phase under a DC electric field.^16^ The flow profile generated by EO is close to the “piston type”, which theoretically can improve the microscopic displacement efficiency. On the other hand, EO may accelerate fluid transport along the dominant channels, leading to premature water channeling and lowering the macroscopic sweep efficiency (Figure 1, (a) is pressure driven (aqua pura), (b) is pressure driven (brine), (c) is electric field driven (brine), and (d) is pressure + electrically driven (brine)).^17^ The magnitude and direction of the electroosmotic effect depend on the Zeta potential (affected by pH, ionic species and concentration), electric field strength, fluid viscosity and dielectric constant, as well as the permeability and tortuosity of the porous medium.^12^ The content and type of clay minerals in the reservoir have a significant effect on electroosmotic flow and are key determinants of the effectiveness of EEOR.^12^ Chilingar et al. used synthetic sandstone and Berea sandstone to study the effect of DC on permeability and related flow characteristics of sandstone and showed that the flow rate increases with the increase of potential gradient and there is a “hysteresis effect”, i.e., the flow rate is still higher than the initial flow rate after treatment.^18^ Their study also showed that the EOF rate increases if one or more of the following occurs: (1) an increase in zeta potential, which is caused by a decrease in the ionic concentration or valence, (2) an increase in the applied potential, (3) a decrease in the resistivity coefficient of the formation.^18^ X-ray analysis in this study showed a substantial reduction in the lamellar structure of the clay after the application of DC.^18^

**Figure 1:**
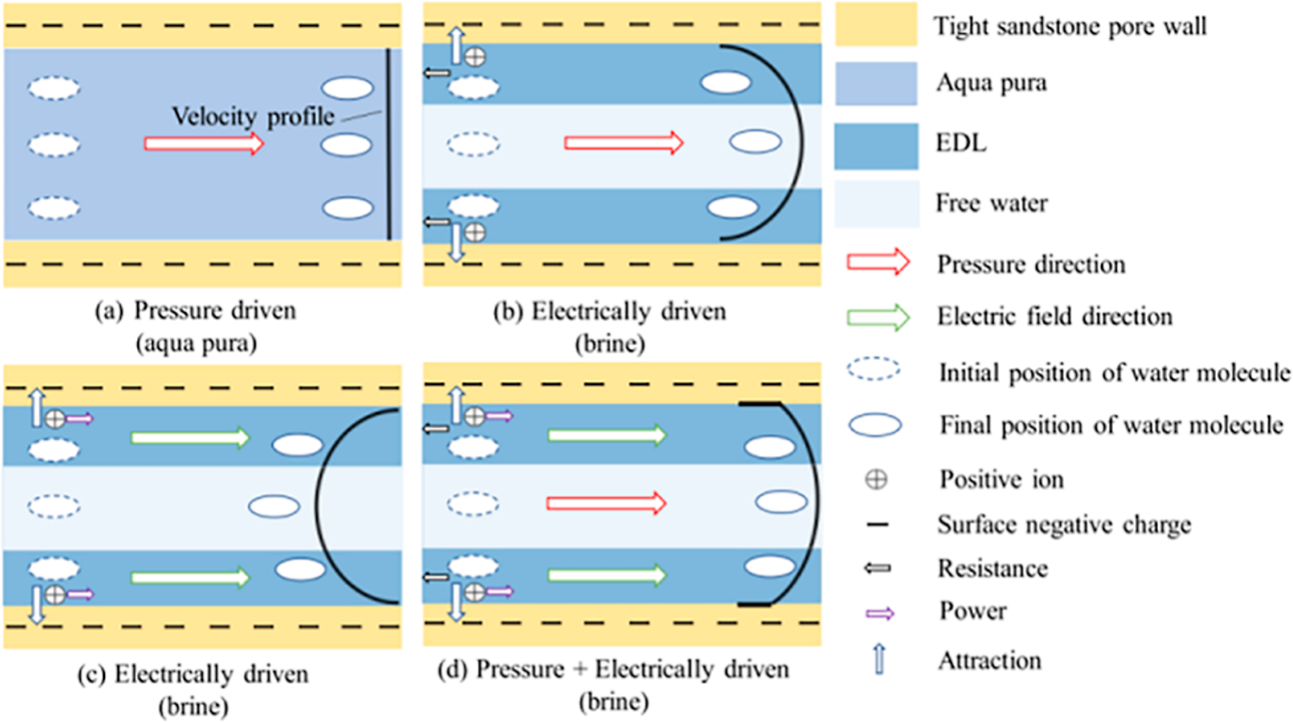
Schematic diagram of pore flow at different electric field energies, (a) is pressure driven (aqua pura), (b) is pressure driven (brine), (c) is electric field driven (brine), and (d) is pressure + electrically driven (brine).^17^ Reprinted with permission from ref 17. Copyright 2023, Energy & Fuels.

Electrophoresis (EP) refers to the movement of charged particles or droplets (e.g., oil droplets, clay particles, asphaltene micelles, injected nanoparticles, etc.) dispersed in a pore fluid relative to the fluid under an electric field.^19^ To understand the role of EP in EEOR, it is first necessary to clarify the electrostatic mechanism of various types of particles in reservoirs. The charging mechanism of crude oil droplets: In typical reservoir water environments, crude oil droplets are typically negatively charged.^20^ The main reason why crude oil droplets carry negative charges is that the natural surfactants contained in crude oil, such as acidic components like naphthenic acids, undergo dissociation at the oil–water interface, forming negatively charged carboxylate ions (R–COO−). Additionally, anions in formation water, such as Cl^–^ and SO_4_^2–^, may also adsorb at the oil–water interface, further enhancing its negative charge.^20^

The charging mechanism of asphaltenes and resins: as the most polar components in crude oil, the surface charges of asphaltenes and resins originate from the protonation or dissociation of functional groups containing heteroatoms such as nitrogen, oxygen, and sulfur in their molecular structures.^21^ The net charge of asphaltic aggregates can be either positive or negative, depending on the nature of the surrounding solvent, pH value, and the presence of stabilizing gum molecules, making their behavior highly complex.^22,23^

The charging mechanism of clay particles: as mentioned earlier, clay minerals (such as kaolinite and montmorillonite) typically carry permanent negative charges due to isomorphous substitution in their crystal structures.^24^

The primary role of EP in improving recovery rates is to mobilize residual oil droplets bound by capillary forces. After water flooding, a large amount of residual oil is trapped in narrow pore throats in the form of discontinuous oil droplets or oil ganglia. This phenomenon is known as the Jamin effect (The Jamin effect refers to the phenomenon where a droplet entering a pore throat deforms, resulting in a smaller diameter at the advancing side than at the trailing side, which creates a higher capillary pressure at the front than at the back, hindering the droplet’s passage through the throat.).^25,26^ This helps to mobilize trapped oil droplets or alter the pore channels. EP flushes out the clay mineral particles and enlarges the pore throats of the porous matrix, promoting more fluid transport.^27^

In the EEOR process, EOF and EPs do not act independently, but occur simultaneously and influence each other. The net migration velocity of a charged particle (such as an oil droplet) in a pore is the vector sum of the electroosmotic velocity (*v*_eof_) of the fluid and the electrophoretic velocity (*v*_ep_) of the particle relative to the fluid.^16,28,29^ This superposition relationship leads to two completely different dynamic behaviors:

(1) Synergistic effect: when the electrophoretic force is consistent with the direction of the EOF, the migration of particles is enhanced. In this case, *v*_eof_ and *v*_ep_ are superimposed in the same direction, greatly accelerating the migration of these particles toward the production well.^29^

(2) Antagonistic effect: in most reservoir situations, EP and EO are antagonistic, which represents a more complex and common scenario. This is because, in EEOR, most widely used electrode layout scheme is to place the anode (positive electrode) in the injection well and the cathode (negative electrode) in the production well.^15,30,31^ Rock surfaces, crude oil droplets, and clay particles are usually negatively charged.^32^ EOF drives water toward the cathode (production well), while EP drives negatively charged oil droplets and clay particles toward the anode (injection well). In other words, if the EO dominates, oil droplets will still move slowly toward the production well, but their speed will be slower than the surrounding water flow. If the EP is very strong, oil droplets may even theoretically move backward relative to the porous medium. Analyzing this antagonistic relationship reveals that the core role of EP in enhancing recovery may not be long-distance transport, but rather localized, pore-scale “unblocking” or “peeling” effects. For an oil droplet fixed in a pore throat by capillary forces, although its macroscopic net velocity is zero, the electric field continues to exert a persistent electrophoretic force on it. Even if this force is opposite to the direction of the main flow, it is sufficient to cause the oil droplet to deform, alter the oil–water-rock three-phase contact line, and ultimately overcome the constraints of capillary forces, “prying” the oil droplet out of the pore throat. Once the oil droplet is desorbed and enters the flowing pore, it is carried by the more dominant EOF. Although it is still subjected to the “drag” of the opposing electrophoretic force during transportation, it overall moves toward the production well. Therefore, the true “synergy” between EOF and EP does not lie in the consistency of force direction but in functional complementarity: EP peels off oil droplets at the microscopic scale, while EOF provides the primary “transport” driving force at the macroscopic scale. Understanding the interaction between EOF and EP is crucial for accurately predicting and optimizing the actual effectiveness of EOR.

Kokal et al. observed that asphaltenes display electrical properties that depend on the pH, ionic strength, and salinity of the electrolyte. Hosseini et al. used a high-resolution optical microscope to study the effect of electrical currents on asphaltenes on glass micromodels. The results showed that the chemical structure and complexity of asphaltenes affect their aggregation rate in the presence of an electric field.^33^ Based on this theory, Azari et al. experimentally determined the charge on asphaltene colloidal particles. Their study concluded that the electrophoretic mobility of asphaltene decreases with increasing particle size.^34,35^ However, it should be noted that the effectiveness of EP depends on whether the particles carry sufficient surface charge (Zeta potential) in the reservoir environment, and this condition is not always met.

### 2.2 Electrical Heating

The thermal mechanism represents one of the most direct and efficient energy conversion processes in EEOR. When an external electric field is applied to the reservoir, electrical energy is transformed into heat within the formation, resulting in viscosity reduction, improved fluidity, and enhanced oil mobility. EEOR can be regarded as a control engineering problem in practice, but its essence lies in thermal recovery.

This heat generation primarily originates from two processes: Joule heating and dielectric loss heating, occasionally accompanied by inductive heating (IH) in conductive structures such as well casings.

#### 2.2.1 Joule Heating

When current flows through a conductive medium (such as saline water, mineralized rock, or injected electrolytes), electrical energy is converted into heat by resistive dissipation (Figure 2),^36–38^ expressed as

1 $${Q=I}^{2}{R=\sigma RE}^{2}$$

where σ is the electrical conductivity and *E* is the electric field intensity. Yuan et al. provided a systematic review of Joule heating in energy materials, showing that both electrical conductivity and field strength jointly control the heat-generation rate.^38^

**Figure 2:**
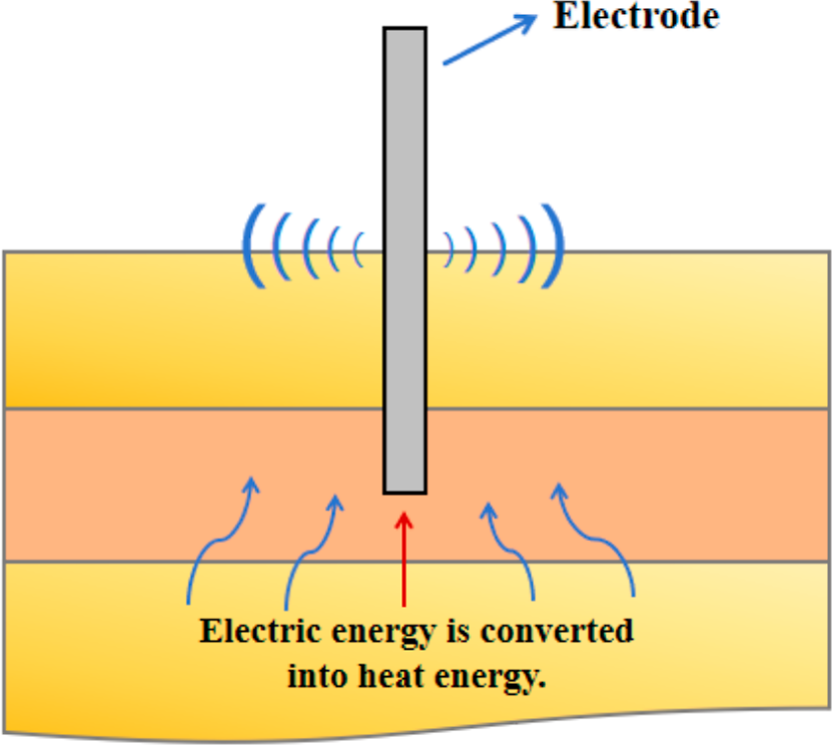
Diagram of the Joule heating process.

Lashgari et al. explored the mechanism through simulation and showed that the rate of heat generation is proportional to the square of the current density and resistivity. The mineralization (which determines the conductivity), water saturation, and temperature of the formation water affect the resistivity and hence the heating efficiency.^36^ Gallo et al. point out that Joule heat is correlated with current density, electric field strength, and bulk conductivity of the porous medium, while changes in temperature alter the rate of electroosmotic flow and affect the rates of migration of ions and chemical reactions, which in turn have an impact on the overall electrokinetic transport process.^39^

Amrouche et al. initiated DC heating using Austin chalk as an oil-wet carbonate reservoir model. The initial implementation of electrical heating resulted in a 27% increase in crude oil recovery rates in seawater and a 16% increase in deionized water. Following the injection of Fe_2_O_3_ nanofluid, recovery rates increased to 32% in seawater and 24% in deionized water. The contact angle and zeta potential decreased from 124° to 36° and from −24.4 to −23.7 mV, respectively, when nanofluid was injected in seawater, leading to better nanofluid stability and penetration into the carbonate rock as shown by increased pellet porosity from 6.6% to 14.8%. This observation was supported by an increase in particle porosity from 6.6% to 14.8%. Moreover, it was found that the interfacial tension was reduced from 72 to 32.7 mN/m in the premagnetized samples with Fe_2_O_3_ NPs injection compared to 33.2 mN/m in the samples with MgO injection. Experimental findings demonstrated that the effect of the generated electric pairs on surface charge is transient, as the rock’s zeta potential immediately reverts to its original value following power disconnection. Laboratory findings indicate that the mechanism of this hybrid EOR technology is based on electrowetting and nanofluid adsorption.^40^

#### 2.2.2 Dielectric Loss Heating

In alternating electric or electromagnetic fields, polar molecules attempt to align with the oscillating field. The orientation lag at high frequencies causes friction and collision between molecules, converting electromagnetic energy into heat. The volumetric power loss can be written as^41,42^

2 $${Q=\omega \varepsilon }_{0}\varepsilon_{r}^{\prime \prime }E^{2}$$

where ω is the angular frequency of the exciting radiation, ε_r_^″^ is the imaginary part of the complex relative permittivity of the absorbing material, ε_0_ is the permittivity of free space and *E* is the electric field strength.

Kwak et al. experimentally demonstrated localized dielectric loss heating in microscale devices, attributing the phenomenon to polarization lag and molecular relaxation.^43^ Yadali Jamaloei further combined laboratory, numerical, and pilot-scale studies, showing that radiofrequency (RF) heating can raise near-wellbore temperatures by over 120 °C and enhance productivity by approximately 30%.^44^ Fanchi simulations demonstrate that the ratio of reservoir electrical conductivity (σ) to dielectric constant (ε′) directly influences near-wellbore temperatures: when σ/ε′ is low (low conductivity, high dielectric), energy is primarily converted to heat via dielectric losses, with temperature increases concentrated in dielectric material zones; when σ/ε′ is large (high conductivity, low dielectric), the proportion of Joule heating increases, but uneven current distribution can easily cause localized overheating.^45^

#### 2.2.3 Inductive Heating (IH)

In a medium-frequency alternating magnetic field, eddy currents are induced in conductive materials (such as steel casing or mineral phases), generating Joule heat without direct electrical contact.^46^

Sherwali et al. simulated electromagnetic induction heating in the Athabasca oil sands and reported that formation temperature could increase by about 100 °C, leading to a predicted recovery improvement of 20–25%.^46^

Alomair et al. conducted laboratory experiments utilizing three unconventional thermal heating methods: namely, electrical resistive electrodes, electromagnetic inductors and microwave heating for EOR. Electromagnetic heating was performed with a sand pack (2 in. by 12 in.) core holder using a crude oil with an API of 17.3 and a viscosity of 540.52 cP. As was documented in the relevant reports, 55 h of heating at 45 °C resulted in the recovery of 51.7% of the oil, while 58.7% was recovered after 47.7 h of heating at 65 °C. However, an increase in the heating temperature to 85 °C resulted in a significant enhancement in oil recovery, reaching 67.8%. In the instance of electrical resistance heating, a recovery of 10.43% oil was achieved after a heating duration of 60 h. As the voltage increased, the heating time decreased. A total of 20.79% of the oil was recovered after 42.4 h of heating. In the context of microwave heating experiments, a range of power levels was utilized, encompassing 10%, 30%, 70%, and 100% of the available power. At power levels of 10% and 30%, the total recoveries were 37.24% and 38.44% for heating times 240 and 230 s, respectively. In contrast, 70% and 100% power levels were able to recover 42.3% and 45.8% in 290 and 270 s, respectively. The average power consumptions were 39, 2570, and 3.775 W h/cc for electromagnetic heating induction, electrical resistance and microwave, respectively. The conclusion drawn was that microwave heating for oil recovery is preferable to other electrical heating methods on the basis of the increased oil recovery and power consumption. It is evident that there is a critical power and heating time for which economic limits must be determined. This task is of crucial importance.^47^

Compared to dominant thermal recovery technologies like cyclic steam stimulation (CSS) and steam-assisted gravity drainage (SAGD), electric heating demonstrates several significant advantages. First, it is a “waterless” technology that conserves precious water resources while eliminating boiler fuel consumption and substantial CO_2_ emissions associated with steam generation, resulting in a smaller environmental footprint. Second, electrical energy is applied directly within the reservoir, bypassing the substantial heat loss associated with long-distance steam transportation through wellbores. This results in higher energy efficiency, particularly in deep or thin-seam heavy oil reservoirs. Overall, electrical heating enhances oil recovery mainly through resistive and dielectric loss mechanisms, transforming electric energy into in situ thermal energy. With advances in electromagnetic technologies, future research focuses on frequency-dielectric optimization, multiphysics coupling (e.g., electromagnetic–dielectric hybrid), and low-carbon, high-efficiency field implementation.

### 2.3 Electrochemistry

Electrochemistry fundamentally involves chemical reactions, which are either driven by an applied potential difference (electrolysis) or generate a potential difference (such as in batteries or fuel cells). Unlike traditional chemical reactions, a key characteristic of electrochemical reactions is that electrons are transferred through an electronic conductive circuit rather than directly between atoms, ions, or molecules.^48^ In EEOR, these electrochemical processes modulate interfacial properties, facilitate ion and particle migration, and drive molecular transformations via electrowetting, electrolysis, and electric field-induced redox reactions.

Electrowetting refers to the alteration of rock surface wettability under an electric field. It is crucial to distinguish its mechanism in reservoir engineering from its more commonly understood application in materials science. In materials science, electrowetting primarily controls the shape and contact angle of a water droplet on a surface.^49^ However, in reservoir rocks, the effect is more profound and involves fundamentally altering the properties of the rock and fluid. The process is driven by a combination of factors: the electric field can alter the surface potential of rock minerals, disrupting the electrostatic bonds that adhere polar crude oil components (like asphaltenes) to the rock surface, leading to their desorption.^50,51^ Furthermore, the field can induce chemical oxidation which increases the density of hydrophilic groups, and physically alter the surface roughness at the pore scale.^52^ These mechanisms collectively drive a wettability shift from oil-wet or mixed-wet toward a more favorable water-wet state, which reduces capillary trapping and significantly improves oil displacement. Karna et al. investigated the mechanism of contact angle change under a DC electric field by molecular simulation. They found that the change in interfacial hydrogen bonding structure and the increase in viscous force in the interfacial layer lead to a decrease in contact angle.^53^ DC electric fields can alter the wettability of rock surfaces through various mechanisms, usually by promoting the transition from oil-wet to water-wet surfaces, which is critical for enhanced recovery.^54^ Zhang et al. investigated the effect of DC voltage on tight sandstone wettability using contact angle tests, Fourier Transform Infrared Spectroscopy (FTIR), and Atomic Force Microscopy (AFM), revealing that electric field-induced chemical oxidation (increased hydrophilic groups) and enhanced surface roughness (enlarged pores) collectively drive wettability changes.^52^ Furthermore, the change of the wetting state from Cassie–Baxter to Wenzel in the presence of DC directly enhances the surface hydrophilicity.^55,56^ They also investigated the surface roughness in the intragranular region (quartz and feldspar dominated) and the intergranular region (clay minerals, microcracks) of sandstone using AFM and scanning electron microscopy (SEM). They found that the migration of clay minerals under the electric field would lead to roughness differences in different regions, which would enhance the hydrophilicity of the rock. Simultaneously, the electric field increases the pore depth. The increase in roughness in the intergranular region is attributed to the electric field increasing the pore length while decreasing the number and depth of pores (Figure 3).^57^ These electrical wetting mechanisms provide a theoretical basis for EEOR. It should be noted that although laboratory studies consistently show that electrowetting can significantly alter wettability, its application at field scale is complex. This effect is essentially limited to the rock-fluid interface, and compared to mechanisms such as EOF or Joule heating that affect macro-scale fluids, its contribution to the overall oil recovery process may be relatively small.

**Figure 3:**
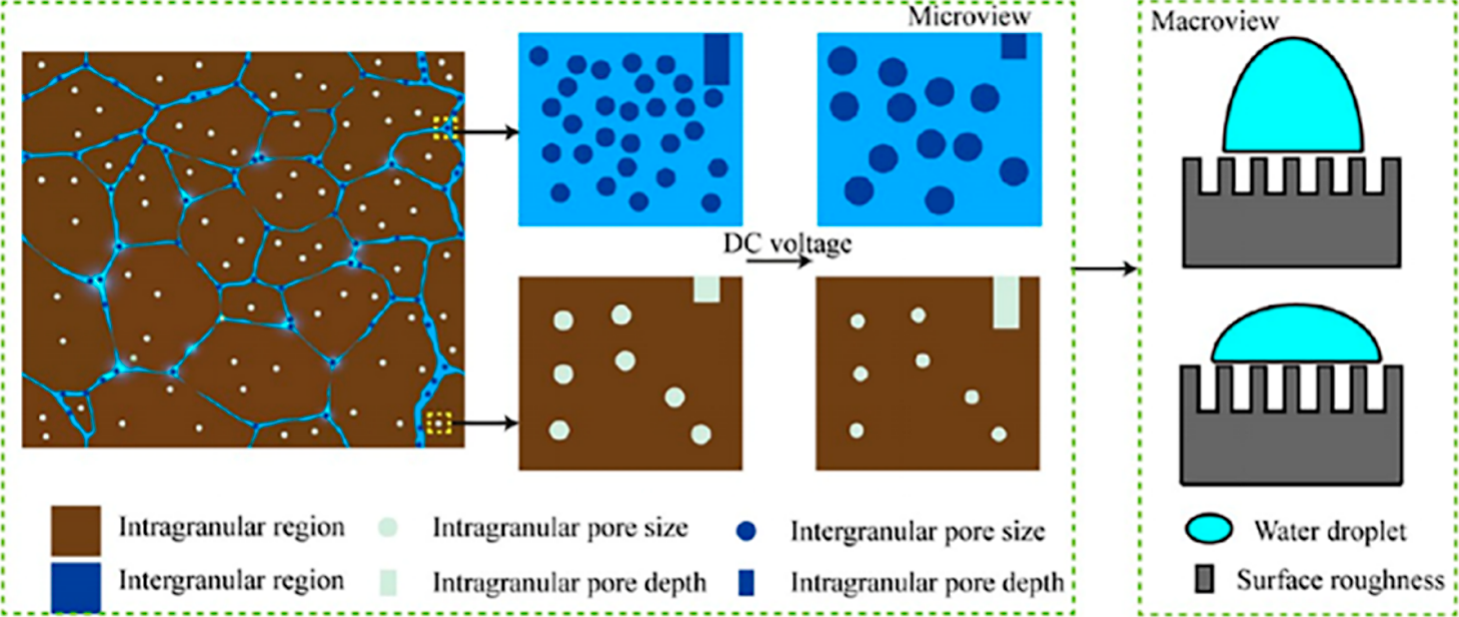
Schematic diagram showing the pore changes in two regions due to electric field exposure.^57^ Reprinted with permission from ref 57. Copyright 2023, Energy & Fuels.

Electrolysis occurs when the applied electric field is strong enough to drive nonspontaneous chemical reactions, primarily at the electrode surfaces. In the context of EOR, these processes typically involve Faradaic reactions occurring at the electrode surface.^58^ A primary example is water electrolysis, which produces H_2_ at the cathode, O_2_ at the anode, and is accompanied by local changes in H^+^ and OH^–^ concentrations.^48^ When an electric field is applied using inert electrodes, the cathode region (water injection well) undergoes electrolysis to produce H_2_ and OH^–^. OH^–^ reacts with organic acids in crude oil to form surfactants that reduce the interfacial tension between oil and water, thereby decreasing oil flow resistance and improving displacement efficiency and oil recovery. This is a potential reason for the decrease in water cut and water phase permeability and the increase in oil recovery observed in refs 59 and 60 In addition to these basic water reactions, redox reactions are also crucial in EEOR, as these reactions can reduce heavy, long-chain petroleum hydrocarbons into shorter-chain compounds, thereby achieving in situ upgrading.^61^ Furthermore, the electric field can induce crude oil suspended particles to aggregate into short chains, thereby reducing viscosity anisotropy. Tao et al. investigated the viscosity-reducing mechanism of paraffin-based crude oil after electric field treatment. They found that the electric field polarizes nanoscale wax particles, causing them to aggregate into streamlined short chains aligned along the flow direction. At −3.1 °C, the crude oil flow rate increased from 2.08 mg/s to 11.66 mg/s (a 460.6% increase), corresponding to a viscosity reduction from 261.3 to 46.7 cP. This treatment effectively reduced viscosity along the flow direction while suppressing turbulence and reducing energy dissipation.^62^

No single EEOR mechanism operates independently; rather, EEOR functions as a coupled multiphysics system where the final outcome is an emergent property of complex interactions. The combined effects of these complex mechanisms can be visualized and summarized in Figure 4.

**Figure 4:**
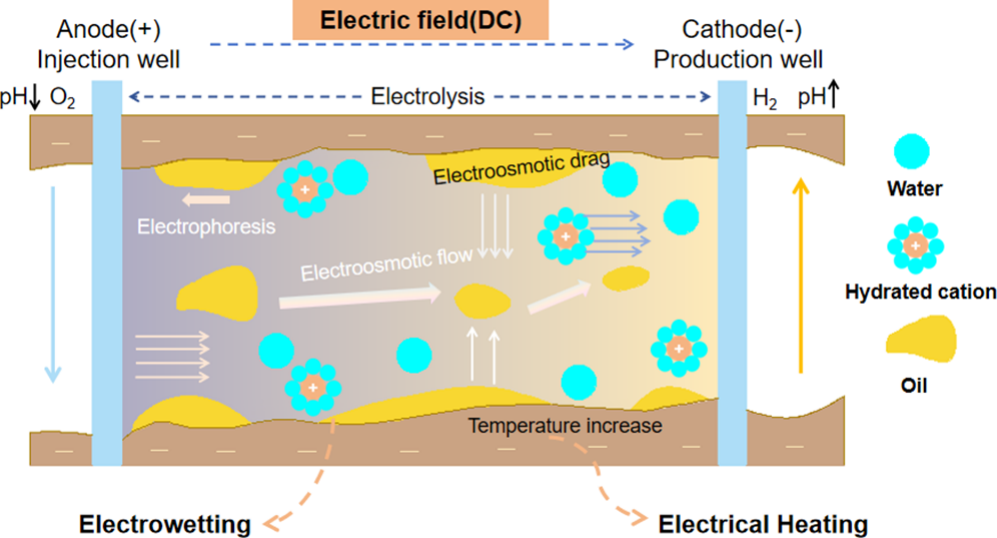
Schematic diagram of EEOR mechanism.

Through comprehensive analysis, the primary and secondary relationships and the synergistic interplay between mechanisms are illustrated as shown in Figure 5. This diagram elucidates the comprehensive pathway from the initial external electric field input to the ultimate improvement in oil recovery. The process unfolds primarily through three parallel core mechanisms: electrokinetic effects, electrothermal effects, and electrochemical effects. Each primary mechanism cascades into a series of secondary phenomena: electrokinetic effects drive macroscopic fluid flow and charged particle migration via EOF and EP; electrical heating, primarily through Joule, dielectric, and inductive heating, elevates reservoir temperature to substantially reduce heavy oil viscosity and induce fluid thermal expansion. Additionally, electrochemical effects, through electrolysis and electrowetting, modify interfacial pH, generate in situ chemical drivers, and enhance rock wettability.

**Figure 5:**
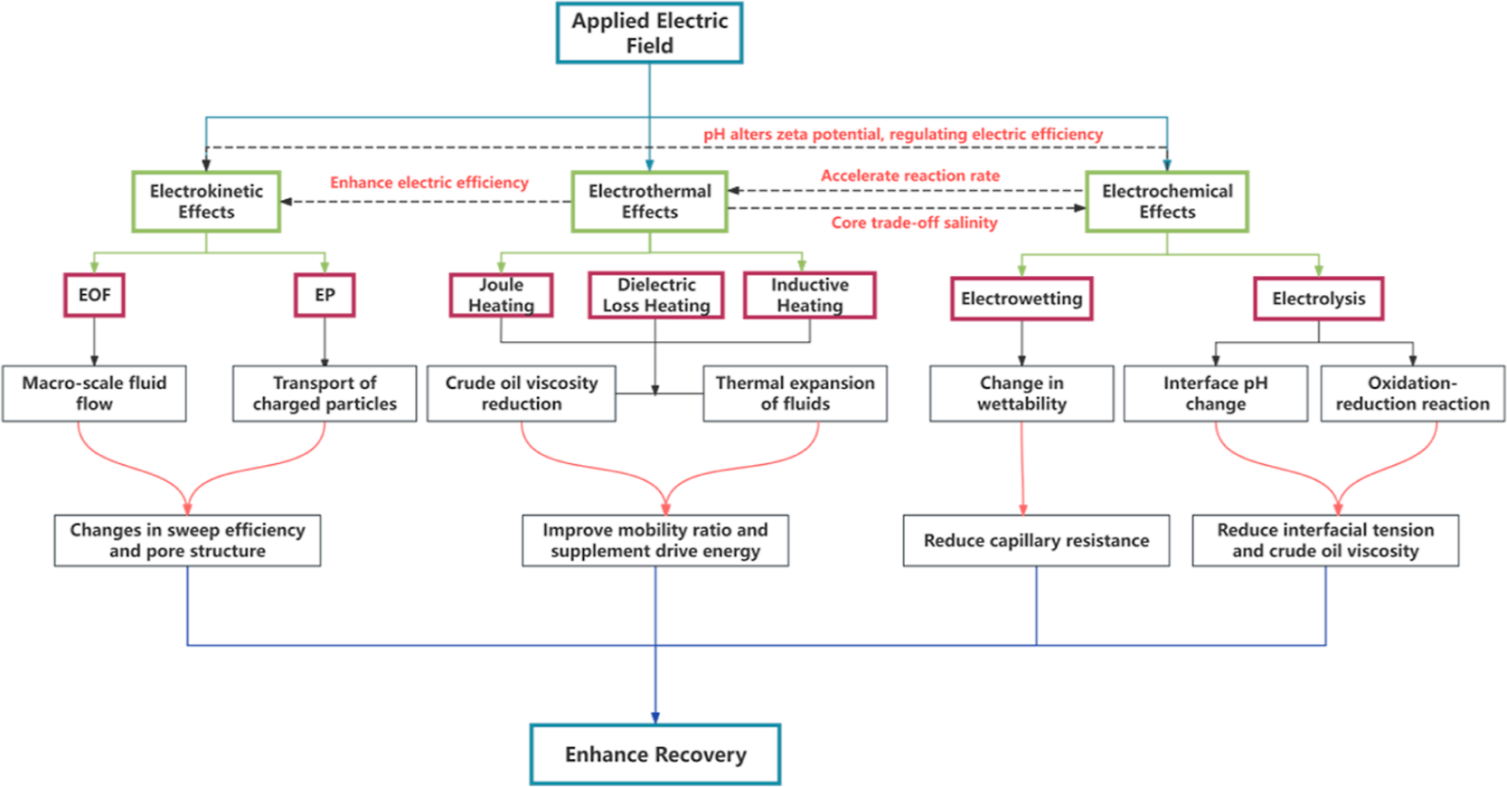
Conceptual framework of EEOR mechanisms and their synergistic interplay.

Crucially, the framework highlights the pivotal synergistic interactions among these mechanisms. The temperature rise from electrothermal effects not only accelerates electrochemical reaction rates but also boosts electrokinetic efficiency by lowering fluid viscosity. Conversely, pH alterations from electrochemical processes can modulate the zeta potential in electrokinetic dynamics. Salinity plays a central role in balancing electrothermal and electrochemical reactions, as elevated mineralization intensifies electrothermal effects under constant voltage conditions; however, excessive salinity may suppress effective electrochemical processes. These interconnected pathways collectively enhance macroscopic sweep efficiency and microscopic displacement efficiency, thereby elevating overall recovery rates. This conceptual framework offers a holistic perspective for comprehending and optimizing the multifaceted processes in EOR.

It is worth noting that a fundamental characteristic of the EEOR system is the complex, mutually constraining antagonistic relationship between its key mechanisms. 1. The conflict between the electrothermal mechanism and the electrodynamic mechanism is primarily governed by the precise control of reservoir brine salinity: higher mineralization in reservoir brine enhances water conductivity. According to Joule’s law, high conductivity facilitates current flow, generating intense Joule heating effects that significantly benefit heavy oil viscosity reduction. However, high salinity strongly compresses the thickness of the EDL at the rock-fluid interface, which is the physical foundation of the EOF. EDL compression significantly weakens or even suppresses the electromotive effect. Conversely, using low-mineralization water expands the EDL and may increase the zeta potential, enhancing EOF generation to provide a nonpressure driving force in low-permeability reservoirs. However, this reduces fluid conductivity, substantially diminishing the Joule heating effect and hindering effective reservoir heating. 2. Within micropores, the final migration of charged oil droplets results from the combined effects of EOF and electrophoretic EP. These forces typically act in opposite directions. Although the functional complementarity between EOF and EP (EP responsible for “detachment” of oil droplets, EOF for “macroscopic transport”) is widely accepted in academia, most research remains at the qualitative description level. Therefore, salinity represents a core trade-off parameter in designing EEOR schemes. Conditions favorable to one mechanism are often detrimental to the other.

## 3 Factors

The effectiveness of EEOR is controlled by a number of factors, which fall into two main categories: electrical parameters and reservoir/fluid properties.

### 3.1 Electrical Parameters

#### 3.1.1 Voltage (V)

Voltage is an important parameter to generate electroosmotic flow and Joule heat. Typically, as voltage increases, Joule heating and electrokinetic effects improve, enhancing the oil recovery. However, an optimal voltage exists, beyond which recovery efficiency may decline due to adverse effects such as water channeling caused by excessively rapid EOF.^17^ As demonstrated by Chilingar et al. and discussed previously, increasing the applied potential enhances the electroosmotic flow rate.^18^

#### 3.1.2 Current (*I*) and Frequency (ω)

These parameters collectively determine which EEOR mechanisms are activated, particularly the distinction between thermal and electrokinetic effects.

Current (*I*): this is the key parameter for Joule heating. According to Joule’s law (eq 1), the current directly determines the rate of heat generation and also affects the rate of electrochemical reactions. The current itself is controlled by the applied voltage and the reservoir’s electrical conductivity.

Frequency (ω): this parameter is critical for the other two electrothermal mechanisms. Dielectric loss heating is directly proportional to the angular frequency (eq 2) and is therefore dominant at high frequencies (e.g., RF).

IH relies on eddy currents generated by medium-frequency alternating magnetic fields.

#### 3.1.3 Electrode Material and Spacing

The material, position, and spacing of the electrodes determine the distribution pattern and intensity of the electric field in the reservoir, which directly affects the current path, the heating area and the scope of action of the EK effect, and has an important impact on the overall efficiency.^63,64^

#### 3.1.4 Type of Electric Current

DC establishes a continuous directional electric field in a porous medium. This electric field drives the ions in the pore fluid to undergo directional migration and drives the fluid as a whole to undergo directional flow. The directional flow produced by this electrodynamic effect is the central mechanism of many EEOR-based applications. The direction of AC changes periodically, and while the AC can also cause oscillations of ions and reciprocal motion of the pore fluid on a tiny scale, the net directional migration and electroosmotic flow are very small over a complete cycle, making it difficult to achieve long-distance directional transport of fluids and ions on the reservoir scale. Therefore, in the field of EOR technologies, DC technology is mainly used to achieve the directional transmission of ions and fluids, while AC is mainly used to enhance the thermal effect.

### 3.2 Reservoir and Fluid Properties

#### 3.2.1 Formation Water Mineralization

Formation water mineralization directly affects electrical conductivity, which determines the magnitude of current and the thickness of EDL.^65,66^ Highly mineralized water conducts electricity well and facilitates Joule heat generation, but may also compress the EDL, attenuate the EK effect, and increase the risk of salinization near the electrodes.^13^ Low salinity water is more conducive to EDL expansion and changes rock surface wettability.^67,68^ The relationship between differential pressure (Δ*P*) and resulting electrokinetic voltage (Δ*V*) is given by the Helmholtz–Schmoluchowski equation:^12^

3 ΔV=C·ΔP

where *C* is referred to as the streaming potential coefficient. The streaming potential coefficient has been studied in the context of other geophysical methods of exploration such as self-potential and streaming-potential; typical values for various rock types range from −12 to 350 mV/atm^13^. In the classical Helmholtz–Schmoluchowski equation, *C* is independent of any microstructural (pore) parameter and is given by

4 C=εζ/ησ

where ε is the fluid dielectric constant, ζ is the zeta potential (associated with the electric double layer), η is the fluid dynamic viscosity, and σ is the fluid conductivity. This relationship assumes (i) the pore hydraulic radius (*m*) is much greater than the EDL thickness, (ii) the flow is laminar (for large *m* we expect nonlinear/turbulent flow), and (iii) the surface conductance is small (≪pore fluid volume conductance). In situations in which electrical surface conduction becomes significant (e.g., decreasing pore size, low conductivity (fresh) fluids) the streaming potential is modified as

5 $$C=\varepsilon \zeta /\eta /\left(\sigma +\sigma_{sc}\right)$$

The Helmholtz–Smoluchowski equation describes the relationship between EO and zeta potential and fluid mineralization.

Zhang et al. investigated the effect of different concentrations of brine on oil recovery efficiency at 10 V and found that the oil recovery efficiency does not increase with the increase of brine concentration, but there exists an optimal value (0.7 mol/L), below which the oil recovery efficiency increases with the increase of concentration, and above which the oil recovery efficiency decreases with the increase of concentration (Figure 6).^69^ EEOR is effective for high viscosity heavy oil.^37^

**Figure 6:**
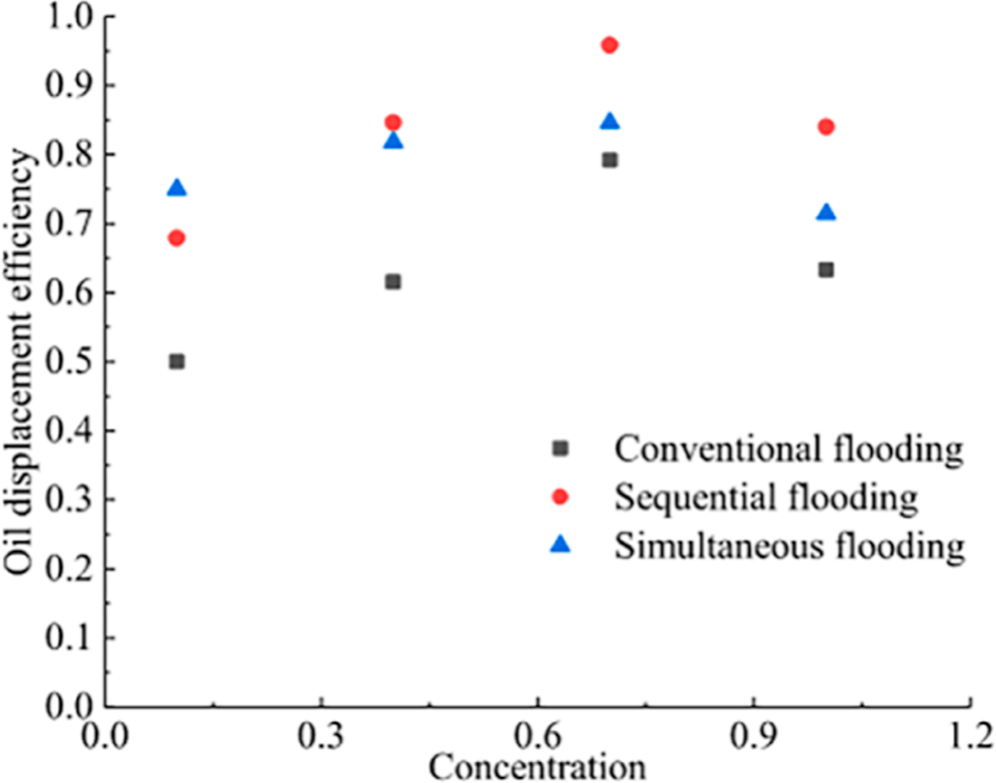
Relationship between oil displacement efficiency and brine concentration for different driving methods.^69^ Copyright 2019, Journal of Petroleum Science and Engineering.

Alomair et al. conducted laboratory experiments comparing media with different conductivities (brine, deionized water). They found that media with moderate conductivity (0.1–1 S/m) and a high imaginary part of the dielectric constant (ε″) exhibited optimal energy absorption efficiency, achieving recovery rates over 30% higher than those under extreme conductivity conditions.^47^

#### 3.2.2 Rock Type and Mineral Composition

Rock type affects its surface chemistry and response to electrochemical reactions. The type, content and distribution of clay minerals have a decisive influence on the ζ potential and EOF, which is one of the key factors in EEOR. Sandstone reservoirs generally exhibit higher recovery than carbonate reservoirs.^13^ This may be related to the different surface charge characteristics, pore structure, and differences with the injected fluids between the two. As mentioned earlier, the electric field causes swelling clays to dehydrate and shrink. This effect is why EEOR is more effective for montmorillonite than kaolinite and Illite in driving oil. In addition, clays may be involved in electrochemical reactions.^15^ Ghosh et al. investigated the effect of DC application direction on the permeability of sandstone reservoirs and oil recovery. The study found that under forward electric field conditions (where the electric field direction aligns with the water flow direction), brine permeability increased by 59%, and additional recovery rate reached 11.6%. This is because the electroosmotic flow aligns with the water flow direction, promoting the decomposition of clay structures into colloidal suspensions that are carried out with the fluid, thereby clearing pore throats and significantly enhancing permeability as well as increasing crude oil mobility. Under reverse electric field conditions (where the electric field direction is opposite to the water flow direction), the brine permeability increased by only 10%, and the additional recovery rate was only 2.1%. This is because the clay in the alkaline environment formed by the reverse electric field is more stable and shows no significant migration.^70^ Another study also demonstrated that a forward electric field significantly increases overall flow velocity by generating an additional electroosmotic flow component, which aids in the main flow process.^18^

#### 3.2.3 Permeability and Porosity

EO provides a fluid driving force independent of the pressure gradient and is therefore particularly effective in tight reservoirs with very low permeability, significantly reducing the differential pressure required for displacement.^12^ Ikpeka et al. state that EEOR is more effective in tight reservoirs with permeabilities of 0.5 mD to 1.5 mD. This specificity makes EEOR not intended to be a full displacement for conventional EOR technology, but rather an important complementary tool for attacking specific types of hard-to-recover reservoirs.^13^

#### 3.2.4 Other Factors

In addition to the above electrical parameters and reservoir and fluid properties, other factors also affect the electric field’s ability to drive oil, such as the distribution of wetted oil and water, temperature and pressure, and the type of displacing fluids, which can have complex interactions with the electric field. These factors affect the EP, wettability, IFT, and overall effect, which in turn affects the oil displacement efficiency.

In conclusion, the effect of EEOR is the result of the coupling of various parameters, which involves numerous factors and complex processes (its main influencing factors are shown in Table 1). These influencing factors provide a detailed reference when screening EEOR reservoirs. Therefore, detailed petrophysical, hydrochemical, and geological characterization of the target reservoir can be evaluated to optimize the electrical parameters and injection strategy before application. Furthermore, the dominant EOR mechanisms vary depending on the reservoir archetype, creating a predictive framework for application screening.

**Table 1:** Summary of Key Factors Affecting EEOR Effectiveness

factor	impact on EOR
electrical parameters	
voltage	enhanced drive and Joule heating, but too high increases energy consumption/risks
current and frequency	the current and frequency affect the degree of heating
type of electric field	DC is more commonly used in EOR; AC usually provides Joule heating effect
electrode configuration and spacing	Determines electric field distribution and current path; needs to be designed according to reservoir characteristics
reservoir/fluid properties	
rock and mineral composition	rocks affect surface charge, EDL; sandstones usually superior to carbonates; clays affect electroosmotic flow, ion exchange, electrical conductivity; montmorillonite effects superior to kaolinite, Illite
permeability and porosity	particularly effective in low-permeability reservoirs (as its driving force is independent of pressure gradient); pore structure affects current path and fluid transport
stratigraphic water mineralization and ionic composition	mineralization affects electrical conductivity and electroosmotic flow efficiency; ions affect wettability; optimal mineralization exists

For heavy oil reservoirs, the electrical heating is undoubtedly the dominant mechanism. Viscosity reduction is the primary objective, with other effects playing a secondary role. In fact, electrochemical modification often synergizes with heating effects.

For light oil in tight reservoirs, EOF and EP are critical. The core challenge is establishing flow in low-permeability media, and EOF provides a unique solution for this.

For mixed-wet carbonate reservoirs, electrochemical effects may play a more significant role. Altering wettability and the in situ generation of surfactants may be more critical than macro-fluid-driven mechanisms.

## 4 Hybrid Methods and Advanced EEOR Technologies

### 4.1 Electrically Assisted EOR Techniques

To fully exploit the advantages of EEOR in enhancing fluid flow and wettability, and thereby achieve higher oil recovery under complex reservoir conditions, EEOR is often combined with other methods, mainly including low-concentration acid EOR, “smart water”/low-salinity water EOR, surfactant EOR, thermal EOR, and nanoparticle EOR, all assisted by an applied electric field.

#### 4.1.1 Electrically Assisted Low-Concentration Acid EOR

This technology leverages the electric field’s electroosmotic effect to deliver acid deeper and more uniformly into the formation matrix, overcoming the limitation that conventional acid stimulation only works in the near-well zone. Ansari et al. conducted oilfield acid stimulation by introducing an electric field. The results showed that this method improved displacement efficiency by 30% and permeability by 55% compared to conventional acidizing. Furthermore, the electric field was found to delay acid absorption in the formation, which increased the depth of acid penetration and the resulting flow rate.^71^ Other potential benefits of combining EEOR with low-concentration acidification include reduced interfacial tension, altered wettability, enhanced capillary number, improved sweep efficiency by targeting unswept zones of the reservoir through EP and EO, and enhanced depth of penetration. Previous studies by the same group have focused on estimating the displacement efficiency, permeability enhancement, and minimum current density required to produce oil from cores.^72^

#### 4.1.2 Electrically Assisted “Smart Water”/“Low-Mineralization Water” EOR

“Smart water”/“low-mineralization” water improves recovery. The core of recovery enhancement lies in modulating the chemical composition and mineralization of the injected water and triggering favorable rock-fluid interactions, such as promoting wettability to water-wetness and reducing interfacial tension.^73,74^ The electric field can provide additional driving force for smart water and may further enhance its interaction with the rock surface through ion migration. Yim et al. investigated the electric-assisted hybrid smart water drive for EOR in carbonate reservoirs, and found experimentally that optimizing the seawater ion concentration enhances the drive effect more than dilution alone, and that this method, when combined with the designed low-salinity smart water and applying optimal electric current, could enhance recovery, but there are limitations such as gas generation and salt precipitation.^51^

#### 4.1.3 Electrically Assisted Surfactant EOR

The main function of surfactant is to reduce the interfacial tension (IFT) between oil and water and improve the efficiency of oil displacement. The role of the electric field is to (1) improve the sweep efficiency and transport of surfactant solutions in the formation through mechanisms such as electroosmotic flow, (2) if the surfactant molecules or formed micelles are charged, the electrophoretic effect may also be involved in their transport, and (3) the electric field itself may affect the adsorption behavior of surfactants at the interface. Liu et al. used molecular dynamics simulations to investigate the effects of DC electric field on the adsorption of the cationic surfactant CTAB and anionic surfactant SDS on the mechanism of oil droplet emulsion breaking in oil-in-water (O/W) emulsions. It was found that the electric field induced the oil droplets to move in a fixed direction and agglomerate, and the cationic surfactant CTAB led to faster deformation/agglomeration due to the attraction with the asphaltene at low electric field strength. The anionic surfactant SDS requires a stronger electric field, and the type of surfactant determines the direction of oil droplet movement and emulsification efficiency. This study provides a theoretical basis for the selection of surfactants and optimization of electric field parameters in EEOR.^75^ Surfactant systems can also change the viscosity by altering the interparticle forces through interfacial polarization under an electric field, thus improving the mobility ratio.^76,77^ This effect provides an as yet underexplored avenue for flow control in electric field-assisted surfactant oil drive technology. Wuzhang et al. found that the electrophoretic velocity and direction of oil droplets depended on the type and concentration of the surfactant, that the mobility increased with concentration until the critical micelle concentration (CMC) was reached, and that the absolute value of the electrophoretic mobility increased with the concentration of the surfactant.^78^

#### 4.1.4 Electrically Assisted Thermal EOR

Low-frequency AC or DC electricity is passed into the formation, generating heat due to the electrical resistance of the formation water and, to some extent, the rock matrix.^79^ This method mainly heats the aqueous phase and then transfers the heat to the crude oil through heat conduction and convection. Low-frequency resistive heating relies on conductive pathways, which in reservoirs are mainly formation water, and if water near the electrodes evaporates, it can lead to circuit interruption.^80^ The technology can be used as a stand-alone method or in conjunction with other methods such as steam flooding and solvent injection. The technique is effective in reducing crude oil viscosity and may vaporize water to form in situ steam, DC may also cause in situ cracking/modification of crude oil. Wittle et al. were early advocates and field testers of DC EEOR for heavy oil recovery, where the mechanism also includes in situ cracking.^15^ Adamu et al. revealed the feasibility of RF electromagnetic heating in Nigerian Tar Sands through numerical simulations.^81^ Hiebert et al. found through simulation studies that electrical heating can also be used as a preheat for steam flooding or other thermal recovery methods to improve initial injectivity.^82^ Sahni et al. clarified the mechanism of action of low-frequency resistive heating and high-frequency electromagnetic heating. Low-frequency heating relies on Joule heat generated by the conductivity of the formation water and is achieved by current conduction between the electrodes. High-frequency heating mainly utilizes the rapid rotation of dipoles of polar molecules in the reservoir fluid, which try to align with the rapidly oscillating electric field, causing molecular friction and generating heat.^83^ Yu et al. carried out a numerical simulation study of electromagnetic heating, establishing a multiphysics coupled mathematical model. They considered the effect of temperature on heavy oil viscosity, the start-up pressure gradient of non-Darcy flow, and the dielectric properties of reservoirs. The results showed that this technology can effectively reduce viscosity and the start-up pressure gradient, and improve heavy oil fluidity, which can significantly increase heavy oil production.^84^ Wan compared the effects of air-assisted steam injection, electrically assisted air injection, and recirculating steam injection for heavy oil extraction. They found that air-assisted steam injection was superior to electrically assisted air injection. The key difference is that the synergistic effect of steam heating and air oxidation creates a larger high-temperature combustion zone, whereas electric heating is limited by the decrease in electrical resistance due to high water saturation, and is therefore more suitable for small-scale wellbore decommissioning.^85^ The electrothermal method can reduce the large amount of water required for steam generation, reduce the startup pressure gradient in low-permeability reservoirs, and reduce the heat loss caused by steam flooding in thin layers due to low heat transfer efficiency, and the technology is applicable to thin and low-permeability reservoirs.^84^ DC fields and high temperatures may also lead to thermal cracking of heavy oil and improve its quality. However, there are limitations; while electrical preheating may improve the injectivity and sweep efficiency of subsequent steam flooding, the high power consumption may make it economically unfeasible. Large-scale field applications are limited compared to conventional thermal recovery methods.

#### 4.1.5 Electrically Assisted Nanoparticle EOR

This technique works by dispersing NPs in a fluid and using electric or electromagnetic fields to enhance their transport, stability, and EOR effects. NPs may adsorb at the oil–water interface and reduce IFT, and the electric field may enhance this effect by deforming the oil droplets and increasing the surface area on which the NPs adsorb.^86–90^ In addition, NPs can be adsorbed on the rock surface and change the wettability.^91^ The electric field may affect the transport pattern of NPs, which can block high-permeability channels or pore throats and divert the fluid flow to unswept areas.^92^ Electrodynamic transport seals the location where it occurs, and if the electromagnetic field can direct NPs, especially magnetic NPs, to preferentially seal the high-permeability zone, this could be a powerful way to improve the sweep area. Rahman et al. investigated the effects of SiO_2_, Al_2_O_3_, ZnO NPs, EK, and mixing techniques on EOR for carbonate reservoirs, and the SiO_2_ NPs synergistic with EK enhanced the recovery rate by 15.2–17.6%. The mechanisms include wettability change, interfacial tension reduction and directional seepage.^93^ Shan et al. analyzed the effects of DC and temperature on the adsorption kinetics of perfluorooctanoic acid (PFOA) on activated carbon by modeling and experiments, and found that DC field and low temperature increased PFOA adsorption by 38%. Although it is a non-EOR field, it can still be shown that DC electric fields can significantly enhance the transport and adsorption of substances in porous media, which brings insights for electric field-assisted nanodrive oil.^94^

#### 4.1.6 Comparison of Methods of Electric-Field-Assisted EOR Methods

As shown in Table 2, the EOR mechanism targeted by each hybrid technology varies significantly. These methods leverage the electric field to achieve specific goals, such as extending acidizing treatments into the deeper reservoir, reducing interfacial tension with surfactants, altering wettability with smart water, or blocking high-permeability channels with NPs. Across all these approaches, the electric field acts as a critical “enabler” and “controller”. Whether it is directing NPs, driving acid to greater depths, or transporting heat, it provides a means of controlling the EOR process in a way that is often difficult to achieve in conventional methods that rely on passive transport and reaction.

**Table 2:** Comparison of Electric-Field-Assisted EOR Methods

methods	core synergistic mechanism	key advantages	technology maturity
electric field + low-concentration acid	EOF enables the injected acid solution to travel farther	it achieves higher oil displacement efficiency and enhanced permeability effects	rarely reported, with immature technology
electric field + smart water/low-salinity water	the electric field can provide additional driving force for smart water and may further enhance its interaction with the rock surface through ion migration	modify wettability to regulate rock-fluid electrostatic interactions	most are still in the laboratory or simulation phase
electric field + surfactant	EOF enhances the wetting efficiency and transport effect of surfactants; EP participates in the transport of charged surfactant micelles	electric fields assist surfactants in mobilizing oil droplets, influence their adsorption at interfaces, and alter interparticle forces and the fluidity ratio	surfactant flooding has matured as an independent technology.Its integration with electric fields remains largely confined to laboratory or simulation stages
electric field + thermal recovery	reduce crude oil viscosity and may also cause in situ cracking/modification of crude oil	primarily targets heavy oil to reduce viscosity, mitigate thermal expansion, and improve crude oil quality; can serve as a preheating method for steam flooding	field tests have been conducted in heavy oil reservoirs
electric field + NPs	electric fields cause NPs to block highly permeable channels; electromagnetic fields can directionally guide magnetic NPs	modify IFT and wettability, blockage, catalysis, and enhance thermal effects	rarely reported, with immature technology

However, a clear distinction in technological maturity exists. While surfactant and low-salinity water flooding are mature as standalone technologies, their combination with electric fields—along with the less mature acid and NP hybrids—remains largely in the laboratory or simulation stage. The core challenge facing emerging methods such as nanofluid EOR lies in their stability under high-temperature, high-pressure, and high-salinity conditions, as well as in the presence of electric fields. The notable exception is electrothermal technology, which has been tested at heavy oil field sites. This overall situation highlights a significant gap between laboratory research and large-scale practical deployment.

### 4.2 Advanced EEOR Technologies

#### 4.2.1 Electromagnetic-Assisted Composite EOR Technology

Electromagnetic-assisted composite EOR technology achieves efficient oil production and energy conservation by coupling electromagnetic heating with mechanisms such as chemical agents, solvents, or nanofluids. Unlike conventional steam-assisted thermal recovery, this technology enables volumetric, controllable in situ heating that significantly reduces heat loss and is suitable for deep and heterogeneous reservoirs. The synergistic interaction between electromagnetic energy and functional materials further improves interfacial wetting, reduces viscosity, and expands sweep volumes. Multiple studies indicate that such composite systems hold significant potential for achieving multiphysics field synergy and low-carbon, high-efficiency oil recovery.

Zhang et al. studied extra-heavy oil in the FC block of Xinjiang, China, comparing conventional and enhanced thermal recovery techniques through three sets of 3D physical simulation experiments (SAGD, EH-SAGD, EHES-SAGD). Results indicate that electrical heating synergistic expanding solvent-SAGD (EHES-SAGD) delivers optimal performance: The peak oil production rate reached 14.2 mL/min, surpassing both SAGD (10.5 mL/min) and EH-SAGD (12.5 mL/min). In terms of energy consumption, its cumulative equivalent steam-to-oil ratios (CESOR) was 7.7 and cumulative equivalent energy-to-oil ratios (CEEOR) were 21.6 kJ/mL, lower than SAGD (9.9 and 27.8 kJ/mL) and EH-SAGD (8.6 and 24.1 kJ/mL). Regarding temperature distribution and steam cavities, the EHES-SAGD steam cavity coverage area expanded by 25% compared to SAGD, with residual oil saturation in the swept area reaching 0.13 (SAGD: 0.22). Foam oil formation resulted in a minimum instantaneous water cut of 43%, significantly lower than the final values of the other two methods (95.9% and 94.7%).^95^ The study by McPherson et al. introduced the electromagnetic flood (EMF) concept as a field-scale numerical model for electromagnetic-assisted bitumen recovery in the Athabasca oil sands. Unlike conventional electromagnetic EOR that produces localized near-well heating, EMF employs upper and lower horizontal electrode wells to establish a midfrequency (30–300 kHz) electromagnetic field propagating laterally through the reservoir. The resulting Joule and dielectric heating creates a continuous thermal front between the wells—analogous to steam flooding-enabling deeper, more uniform in situ heating and sustained viscosity reduction. Using physical parameters derived from earlier RF heating experiments, the model predicted that at 250 kHz and 2 MW input power, a 20 m thick oil sand layer could be heated above 200 °C within two years, achieving roughly 45% recovery and an energy efficiency of about 3.25 bbl oil/kWh. Although not a field trial, this work established the theoretical foundation for large-scale wave-propagation-based electromagnetic recovery, bridging the gap between localized RF heating and continuous thermal flooding.^96^

Due to the complex multiphysics coupling involving electromagnetic fields, multiphase flow fields, and temperature fields in the electromagnetic heating process, coupled with the drastic changes in rock and fluid properties with temperature, recent simulation studies have evolved from simple heat conduction models to coupled models that comprehensively account for multiple complex factors. These include the exponential effect of temperature on heavy oil viscosity, the pressure gradient required to initiate non-Darcy flow, changes in reservoir dielectric properties with water saturation and temperature, and heat dissipation through the caprock and floor.^84^

#### 4.2.2 Electromagnetic-Dielectric Coupling EOR Technology

Electromagnetic-dielectric coupling EOR technology enhances oil recovery by combining electromagnetic energy input with the dielectric response of reservoir media. Under alternating electromagnetic fields, polar molecules within the formation undergo polarization and relaxation, converting electromagnetic energy into heat and facilitating viscosity reduction. This coupling mechanism enables efficient in situ heating and improved displacement efficiency compared with conventional thermal or electrical technologies.

##### 4.2.2.1 Electromagnetic Fluid EOR Technology

In recent years, the synergistic application of electromagnetic waves and nanofluids has opened new avenues for enhancing oil recovery (EOR). Hamid et al. systematically investigated the interfacial behavior of Mn-doped superparamagnetic Fe_3_O_4_ nanofluids under electromagnetic fields, revealing that electromagnetic waves can significantly improve oil displacement efficiency by regulating NP polarization and magnetic response. In the study, Helmholtz coils generated uniform electromagnetic fields under direct current (DC) and alternating current (AC) conditions to conduct interfacial tension (IFT) tests on Mn_*x*_Fe_2‑x_O_4_ nanofluids. Results indicated that introducing electromagnetic fields markedly reduced IFT, with AC electromagnetic waves demonstrating greater effectiveness than DC in lowering oil–water interfacial tension, exhibiting stronger dynamic polarization coupling effects. The study further indicates that Mn doping enhances the magnetic saturation intensity and response sensitivity of Fe_3_O_4_. By promoting molecular polarization relaxation and electromagnetic energy absorption, it achieves higher energy conversion efficiency. At a 0.01 wt % concentration, AC electromagnetic waves reduced IFT by approximately 1.2 mN·m^–1^, indicating that electromagnetic waves accelerate oil droplet dispersion and wetting reversal at the microscopic level. Further DFT simulations reveal that Mn atoms preferentially substitute tetrahedral Fe^3+^ sites, enhancing lattice stability and magnetic moment. This macroscopically improves the magnetic response and heat transfer behavior of the nanofluid. Collectively, this study provides a mechanistic foundation for “electromagnetic-nano composite EOR technology”: electromagnetic wave-induced polarization coupled with dielectric loss heat promotes crude oil viscosity reduction; superparamagnetic NPs enhance hysteresis loss and microscopic perturbations in alternating fields, optimizing fluid kinetic energy distribution; doping regulation (e.g., Mn, Co, Zn) increases magnetic susceptibility and energy absorption, making the system more suitable for low-conductivity reservoirs.^97^ Adil et al. conducted systematic experimental studies, successfully implementing electromagnetic field-assisted zinc oxide nanofluid (ZnO nanofluid) EOR experiments, thereby validating the effectiveness of this technology under high-temperature reservoir conditions. The study employed crude oil sourced from the Tapis field, conducting two-phase displacement experiments in a quartz sandstone sand-tube system at 95 °C with permeability ranging from 265 to 300 mD. The experiments incorporated ZnO NPs with particle sizes of 55.7 and 117.1 nm. Under conventional nanodisplacement conditions without an electromagnetic field, the ZnO nanofluid increased OOIP by an additional 8.59–10.27%. When subjected to electromagnetic waves, recovery rates further improved to 9–10.4% OOIP. Results indicate that electromagnetic assistance significantly enhances NP polarization effects and dielectric loss, accelerating oil droplet deformation and wetting reversal processes. This reduces interfacial tension (IFT) between oil and water and improves fluid kinetic energy distribution. Experiments also revealed that ZnO NPs exhibit rotational polarization and electrorheological effects (ER) under alternating electric fields, causing a slight increase in fluid viscosity and improved flowability ratio, thereby enhancing sweep efficiency. As shown in Figure 7, the electromagnetic field-induced droplet deformation mechanism is central to this process: NPs rearrange along the electric field direction under interfacial polarization, forming chain-like structures that increase droplet surface area, enhance adsorption, and reduce interfacial tension. This microscopic mechanism manifests macroscopically as improved recovery rates and stable upward pressure curves.^98^

**Figure 7:**
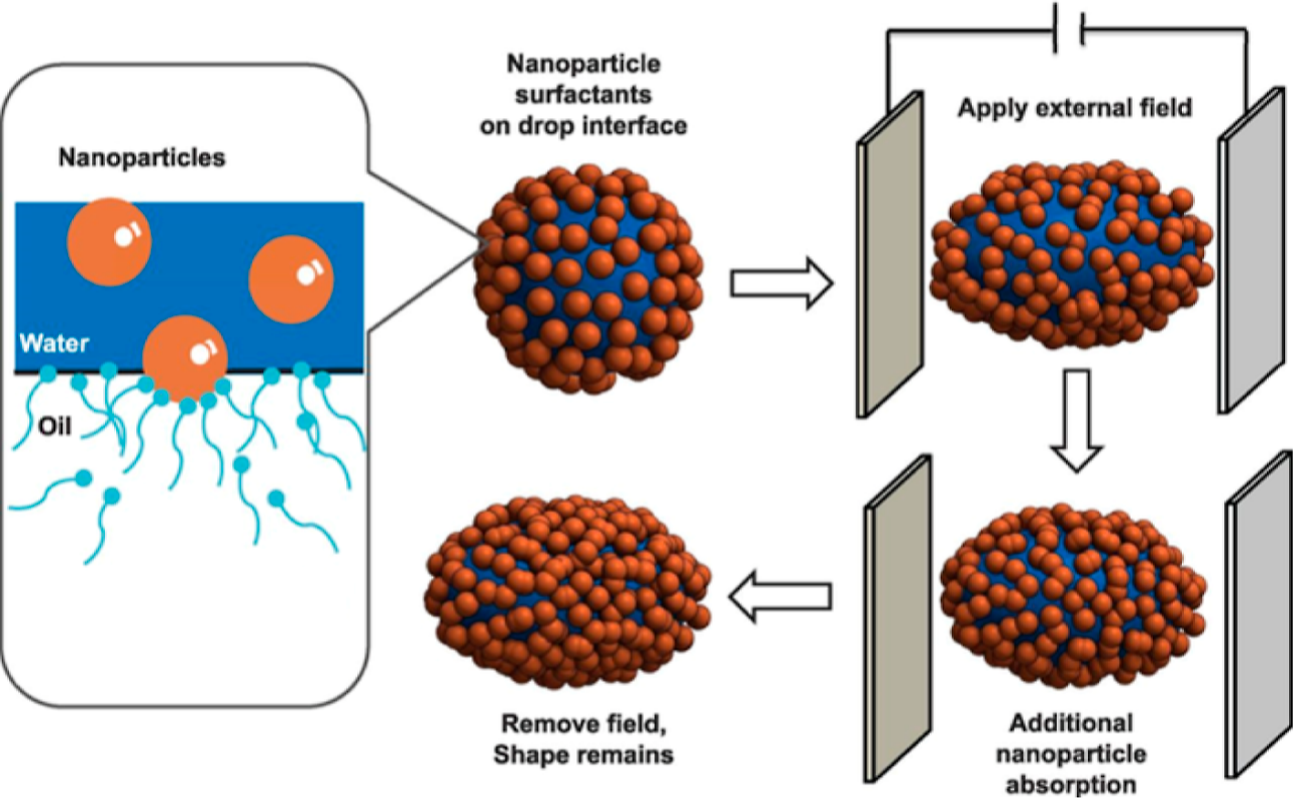
Schematic representation of deformation of oil drop, surrounded with NPs, by an electric field.^98^ The figure is from ref 59, 60 , which is licensed under CC BY 4.0. Copyright 2018, PloS one.

Overall, electromagnetic-assisted nano-EOR technology enhances recovery rates through three synergistic mechanisms: dielectric polarization and dissipation heat effects reduce crude oil viscosity; electrohydrodynamic effects improve fluid flowability ratio and sweep volume; and the formation of NP-polarized chain structures promotes interfacial restructuring and wetting transition. This study demonstrates that Electromagnetic-assisted nano-EOR not only combines the dual advantages of chemical EOR and electromagnetic oil recovery but also achieves higher energy utilization efficiency in high-temperature, low-conductivity formations.^98^ Gharibshahi et al. employed 3-aminopropyltriethoxysilane (APTES), citric acid (CA), and polyethylene glycol (PEG 6000) as surface modifiers to modify Fe_3_O_4_-MWCNT (a nanohybrid material synthesized by coprecipitation of Fe_3_O_4_ NPs with multiwalled carbon nanotubes MWCNT). This was done to validate the effectiveness of the modified nanohybrid in microfluidic EOR. Results indicate that compared to APTES and PEG 6000, citric acid modification enables the nanohybrid particles to maintain a stable, nonprecipitating dispersion in distilled water over extended periods. Furthermore, adding only 0.1 wt % citric acid-modified MWCNT-Fe_3_O_4_ nanohybrids significantly elevated solution temperature. Consequently, after 180 s of microwave irradiation, the water-based solution temperature increased by approximately 11 °C compared to deionized water. Finally, it was found that, by using 400 W microwave radiation, the microfluidic oil recovery increased 24.9%, 30.3%, and 43.9% in comparison with water, Fe_3_O_4_ @ CA, and Fe_3_O_4_-MWCNT @ CA injection without microwave radiation, respectively.^99^ Later, Hasani and Jafari conducted a comprehensive microfluidic study on electromagnetic-assisted magnetic nanofluid flooding for EOR. Superparamagnetic Fe_3_O_4_ NPs were synthesized via coprecipitation and surface-modified with citric acid to improve colloidal stability, while Mn_2_O_3_ NPs were used as a comparative magnetic phase. Flooding experiments were carried out inside a glass micromodel under both the absence and presence of an electromagnetic field (2.45 GHz, 500 W). Without electromagnetic irradiation, surface-modified Fe_3_O_4_ nanofluid (0.3 wt %) increased oil recovery from 19.8% to 35.45%, while under electromagnetic heating, recovery rose sharply to 79.83% (69.87% for Mn_2_O_3_). The improvement was attributed to the magnetic loss mechanism, where NPs absorb microwave energy, generate localized heat, reduce oil viscosity (from 1010 cP to 84 cP), and promote the cracking of heavy fractions into lighter components. The interfacial tension between oil and nanofluid decreased significantly (from 24.8 to 9.4 mN/m), and contact angles indicated a transition from oil-wet to hydrophilic surfaces (up to ∼150°), enhancing oil detachment and mobility. This study demonstrates that the synergistic interaction between electromagnetic fields and magnetic nanofluids triggers simultaneous thermal, interfacial, and wettability modification effects. Due to its higher magnetic susceptibility, Fe_3_O_4_ exhibited superior performance compared with Mn_2_O_3_. The findings confirm the feasibility of electromagnetic fluid EOR, establishing a mechanistic basis for future hybrid electromagnetic-magnetic nanofluid EOR technologies in heavy-oil and low-permeability reservoirs.^100^ Afrooz et al. performed a finite-element simulation to investigate the influence of electromagnetic energy on the oil recovery performance of different nanofluids (CuO, ZnO, and Fe_3_O_4_). A two-dimensional porous-medium model was constructed to simulate a reservoir with an initial oil saturation of 20–40% under a constant temperature of 119 °C and a pressure gradient of 800 psi. Three modes—direct-current potential (DC), DC magnetic induction, and alternating current electromagnetic induction (AC)-were compared. The results (Table 3) showed that the AC mode produced the strongest electric and magnetic fields, enhancing electromagnetic coupling within the porous medium and increasing the recovery factor by approximately 28% (from 22.2% to 28.5%). Among the tested NPs, Fe_3_O_4_ exhibited the highest recovery performance (RF = 28.5%), outperforming ZnO (25.8%) and CuO (17.7%). This improvement was attributed to the formation of magnetized chain structures induced by interactions between the electromagnetic field and magnetic NPs, which enhanced directional migration and interfacial adsorption, facilitating oil detachment from the rock surface. In contrast, dielectric NPs (ZnO and CuO) experienced field-induced polarization, generating transient dipolar chains that increased apparent viscosity and sweep efficiency, thereby stabilizing the displacement front. The mechanism of particle alignment and chain formation under an external electromagnetic field is illustrated in Figure 8. Additionally, hybrid nanofluids (such as ZnO–Fe_3_O_4_ and CuO–Fe_3_O_4_) were evaluated. Although individual nanofluids exhibited slightly higher base recoveries, the application of an electromagnetic field resulted in greater improvements in hybrid systems (4–8% increase). These findings highlight three synergistic pathways of the electromagnetic-nanofluid mechanism: dielectric polarization and Joule heating reduce oil viscosity; magnetic-dielectric chain formation enhances microscale disturbance and wettability alteration; multiphase energy absorption and thermal conduction promote energy utilization and oil mobility. Overall, this study confirms that applying an electromagnetic field during nanofluid flooding significantly improves reservoir hydrodynamics and interfacial behavior, enhancing oil recovery and providing a theoretical basis for multiphysics coupling in electromagnetic-nanofluid EOR.^91^

**Table 3:** Oil Recovery Results for Different Electromagnetic Methods^91^

electromagnetic technique	injection method	initial oil in place (%)	oil in place after 7 days (%)	RF (%)
no electromagnetic	brine + oil + Fe_3_O_4_	40	17.8	22.20
electric potential	brine + oil + Fe_3_O_4_ + electromagnetic	40	17.0	23.00
magnetic induction (DC)	brine + oil + Fe_3_O_4_ + electromagnetic	40	13.0	27.00
electromagnetic induction (AC)	brine + oil + Fe_3_O_4_ + electromagnetic	40	11.5	28.50

**Figure 8:**
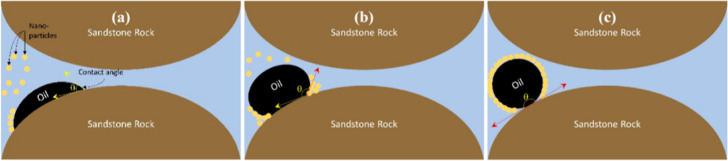
(a) NPs tend to be absorbed to the oil-rock interface of an oil-wet sandstone, (b) absorption of NPs to the oil-rock interface increases the contact angle, (c) while altering the wettability of the rock from oil-wet to water-wet, absorption of NPs to the oil–water interface reduces the IFT and enables oil to mobilize easily.^91^ Copyright 2024, Alexandria Engineering Journal.

These findings indicate that electromagnetic fluid EOR technology holds potential for achieving efficient energy utilization, controllable interface regulation, and environmental sustainability, positioning them as a key future development direction for EOR technologies.

##### 4.2.2.2 Electromagnetic-Other Medium Coupled EOR Technology

Building upon the progress of electromagnetic fluid EOR, where magnetic nanofluids serve as active energy absorbers, recent studies have extended the concept to electromagnetic coupling with other displacement media such as solvents, surfactants, and chemical agents. These hybrid systems combine electromagnetic in situ heating with physicochemical mechanisms to further enhance viscosity reduction, interfacial modification, and sweep efficiency.

Raytheon-CF technology: This is one of the few applications involving “RF + another field”, specifically RF and solvent (CO_2_) synergistic extraction technology. RF + critical fluid EOR technology (RF-CF) represents an emerging unconventional thermal recovery method for oil and gas. This approach involves deploying RF electromagnetic heating devices within formations to rapidly heat reservoirs in situ. Simultaneously, supercritical fluids (such as supercritical CO_2_) are injected. By leveraging the heating effect of electromagnetic fields alongside the fluid’s dissolution and transport capabilities, this method achieves highly efficient recovery. Compared to single thermal recovery, this technology features “heat-solvent” coupling: the electromagnetic field provides uniform and controllable energy to the reservoir, heating the oil layer, reducing viscosity, and promoting hydrocarbon release; the supercritical fluid penetrates pores under high temperature and pressure, dissolving and extracting oil and gas products to form a composite displacement. This synergistic process not only reduces heating energy consumption and formation disturbance but also enables efficient production and fluid recycling. It offers advantages such as lighter oil products, high energy efficiency, and minimal environmental impact. Currently, this technology primarily originates from the RF-CF process developed by Raytheon and CF Technologies, with complete process frameworks outlined in patents.^101^ Recent studies have shown that in heavy oil reservoirs with low thermal and electrical conductivity, conventional RF heating alone suffers from limited heating range and low energy utilization efficiency. To address this limitation, Wang et al. proposed a novel thermal recovery method by combining RF electromagnetic heating with conductive medium injection. In this approach, a conductive material is injected into hydraulically induced fractures to enhance local electrical and thermal conductivity, thereby improving RF heating performance. Numerical simulations and experimental results demonstrated that this method significantly enlarged the heating zone and increased the heating rate at both ends of the antenna, with a maximum temperature rise improvement of up to 3.75 times (Figure 9). The heating effect was highly sensitive to fracture location and electrical conductivity (optimal range: 10–30 S/m), but showed weak sensitivity to specific heat, thermal conductivity, and fracture width. Placing fractures closer to the antenna yielded the highest heating efficiency (Figure 10). This technique avoids the high water consumption and emissions associated with conventional steam-based thermal recovery, offering a high-efficiency and low-carbon heating solution for heavy oil reservoirs, oil sands, and other unconventional resources.^102^

**Figure 9:**
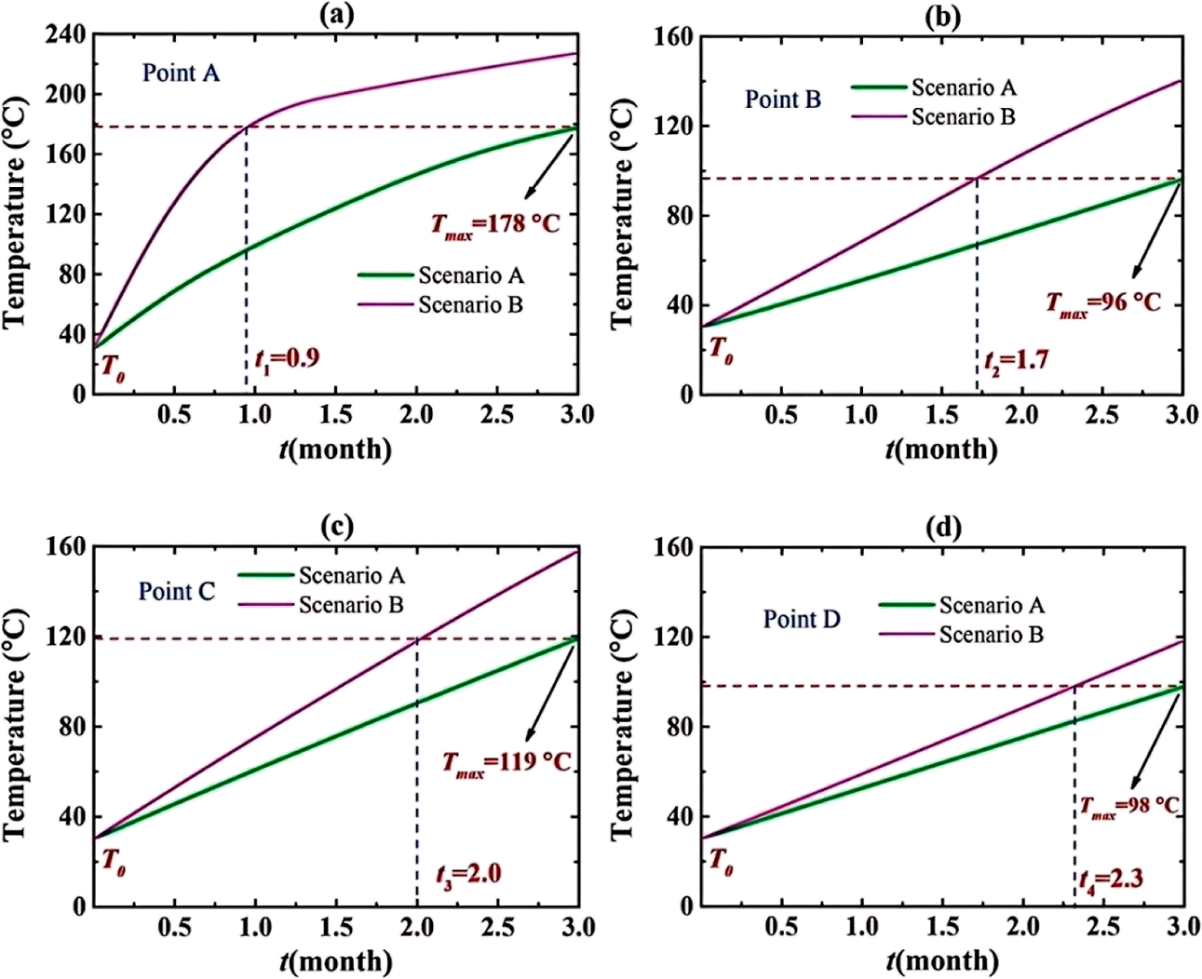
Temperature distributions of four points with time. (a) Point A. (b) Point B. (c) Point C. (d) Point D.^102^ Reprinted with permission from ref 59, 60 . Copyright 2021, Society of Petroleum Engineers. Journal of Petroleum Science and Engineering.

**Figure 10:**
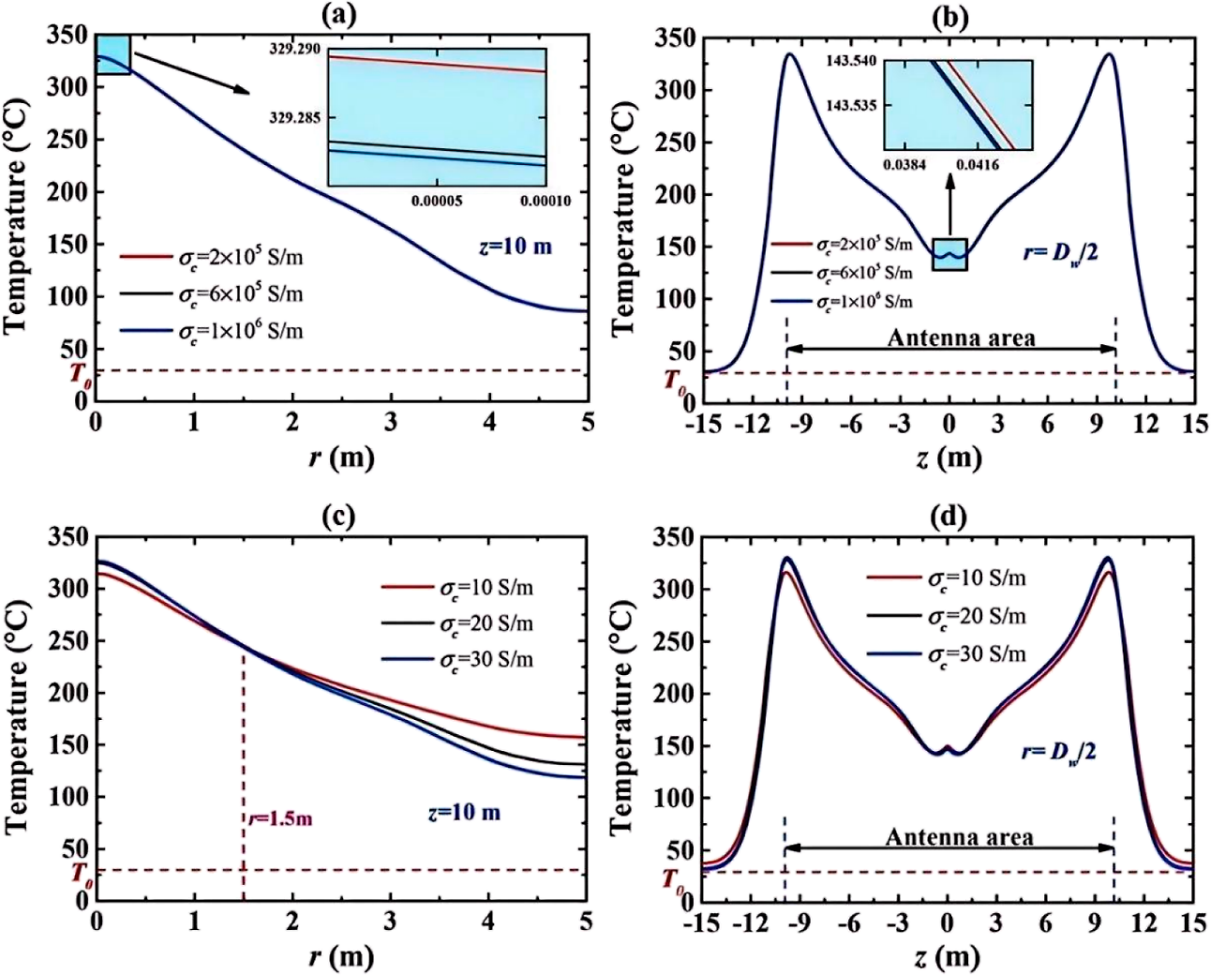
Effects of electrical conductivity of conductive medium on temperature distribution. (a) Radial temperature (2 × 105 S/m ≤ σ*c* ≤ 1 × 106 S/m). (b) Wellbore temperature (2 × 105 S/m ≤ σ*c* ≤ 1 × 106 S/m). (c) Radial temperature (10 S/m ≤ σ*c* ≤ 30 S/m). (d) Wellbore temperature (10 S/m ≤ σ*c* ≤ 30 S/m).^102^ Reprinted with permission from ref 59,60 . Copyright 2021, Society of Petroleum Engineers. Journal of Petroleum Science and Engineering.

#### 4.2.3 Plasma Pulse Technology (PPT)

Plasma pulse technology (PPT) is an innovative approach that utilizes pulsed electric fields and plasma technology to improve oil and gas well productivity. It serves as an alternative to conventional EOR techniques such as hydraulic fracturing (HF), offering lower environmental impact while efficiently enhancing reservoir permeability and fracture density to boost oil and gas recovery rates. The core of this technology lies in utilizing advanced pulsed power systems to store electrical energy in capacitor banks, then releasing it in extremely short durations (typically nanoseconds to microseconds) at exceptionally high peak power levels. The main principle of this technology relies on the application of high-voltage electrical pulses of kilovolts through electrodes inserted into a fluid-filled borehole drilled into a rock specimen. When the electrical potential exceeds the dielectric strength of the medium (commonly water or brine), a rapid electrical breakdown occurs, which results in the formation of a highly conductive plasma channel. This plasma expands almost instantaneously, generating a sharp rise in local pressure and producing strong shockwaves that radiate outward into the surrounding rock. These shockwaves induce high-intensity tensile and shear stresses that exceed the rock’s fracture strength, leading to the initiation and propagation of micro to macro-scale fractures. With the control of the fracturing process via pulse parameter modulation, the technology can provide much lower adverse effects on the environment in comparison to existing traditional methods, such as HF and acid stimulation.^103^ These unique nonthermodynamic mechanical effects enable PPT to be applied not only in EOR for oil and gas wells but also in multiple fields such as geothermal well stimulation, hard rock fracturing without damage, and mineral extraction, demonstrating its broad engineering application potential.

The technology was first introduced to the U.S. industry in 2013 and was originally developed at St. Petersburg State Mining University in Russia. Unlike hydraulic fracturing, where pressurized fluids are used to open or create channels and induce artificial permeability, the PPT generates a high-energy plasma arc that releases intense energy in the form of heat and acoustic waves for a fraction of a second. Subsequently, these impulse waves created removes any clogged sedimentation from the perforation zone, i.e. scale, fines, drilling mud, etc. In addition, the process may lead to the formation of nano- to microscale fractures as a series of impulse waves penetrate deep into the reservoir, thereby enhancing permeability.^104^

Maksyutin and Khusainov selected multiple high-viscosity oil samples and conducted rheological experiments at different temperatures and shear rates. The study revealed that the effective viscosity of the oil samples decreased by an average of 30% after pulsed plasma treatment, indicating that this technology effectively improves the oil’s flowability (Figure 11). It was found that the reduction in the effective viscosity of crude samples was attributed to the destruction of their thixotropic structure. Depending on the composition of crude, their molecular structures can be excited with particular range of frequency.^105^

**Figure 11:**
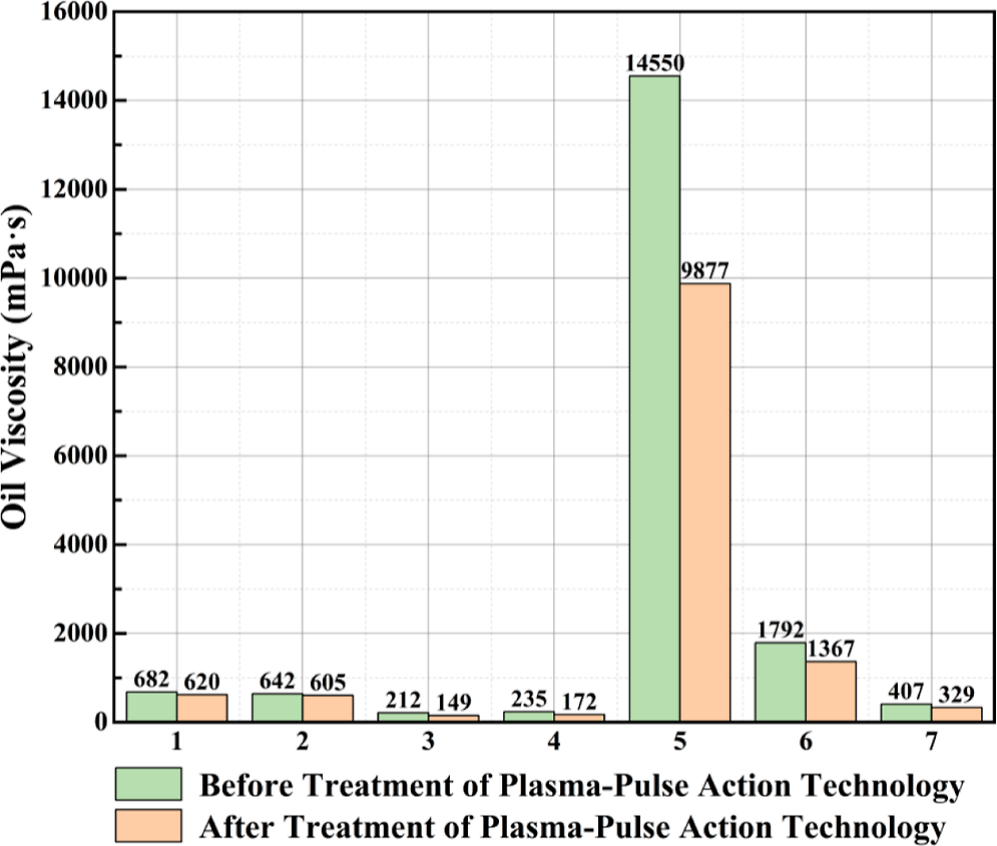
Reduction in effective viscosity observed after application of PPT^105^ (Data sourced from^105^).

Regarding rock porosity, Maddirala et al. evaluated the effects of pulse plasma-based shockwave technology on sandstone porosity and pore connectivity, as shown in Figure 12. Experimental results demonstrate that both porosity and pore connectivity in sandstone samples significantly increase with the number of pulses. Specifically, micro-CT scans of the Berea sandstone sample reveal that the quantity, size, and connectivity of pores and microcracks all enhance as pulse count rises. Ultrasonic measurements indicate a marked decrease in P-wave velocity within sandstone samples following pulse plasma-based shockwave technology exposure. This phenomenon indicates that increased pulsation creates more pores and microcracks, potentially contributing to wave velocity attenuation. The study also calculated a “damage factor”, which progressively increased with pulsation count. This demonstrates a significant rise in rock damage severity and changes in pore structure.^106^

**Figure 12:**
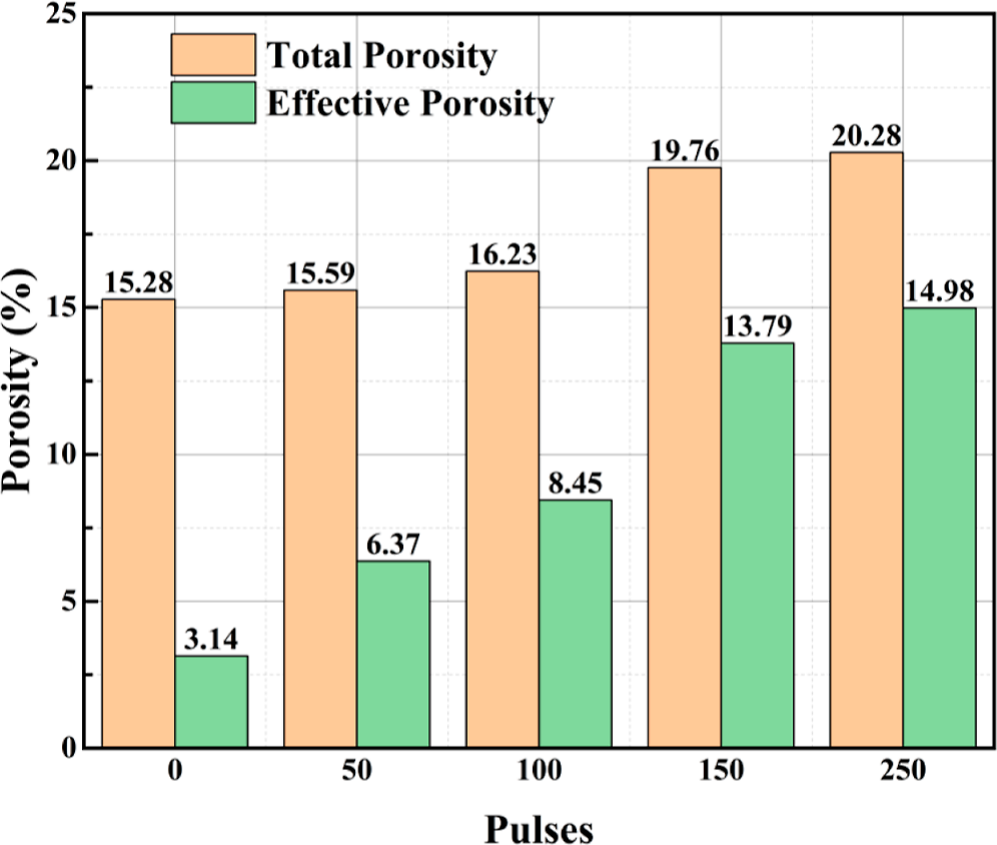
Porosity changes by pulses of plasma-based shockwaves. (Data were taken from ref 106.)

Recent advancements are further reflected in the research conducted by the Soliman team, which demonstrates the use of electrically generated shockwaves via plasma discharge or thermite-induced chemical reactions as a novel means of improving hydraulic fracture propagation and complexity. They investigated plasma-stimulated fracture initiation across multiple materials. They found that aluminum wires produced more extensive fractures due to additional thermite reaction energy. Rock type and heterogeneity strongly influenced fracture morphology: with aluminum wires, fractures were wider and longer, while without wires, fewer but larger fractures formed. In limestone, more directional fractures were observed due to its heterogeneity; whereas in the more homogeneous sandstone, fewer but larger fractures formed. Under confining pressure, higher energy and repeated discharges were required. Permeability measurements indicated a 10 to 100-fold increase in permeability for sandstone.^107^

PPT demonstrates significant effectiveness in enhancing permeability, porosity, and fracture density, and has been proven to increase production in oil, gas, and geothermal wells. Its primary limitations include a short stimulation radius and logistical challenges associated with cable-based delivery systems. Plasma-assisted drilling also represents a promising future direction for integration.^103^

Before delving into the current status of EEOR technology’s field applications, it is essential to systematically review the key researchers and their contributions that have significantly advanced this field, particularly in understanding its core mechanisms. Table 4 categorizes these researchers and their critical findings based on the core mechanisms they studied.

**Table 4:** Summary of Principal Laboratory Experiments of EEOR

number	mechanism	researchers	key findings
1	EO and EP	Chilingar^18^	1. Research revealed the effect of direct current on the permeability of sandstone cores, showing that flow velocity increases with increasing potential gradient and exhibits a hysteresis effect. 2. The EOF effect was found to be significantly greater in clay-bearing cores than in pure silica sandstone, highlighting the role of clay minerals—particularly montmorillonite. 3. X-ray analysis indicated a reduction in clay layered structure following direct current exposure
2		Ghazanfari^16^	proposing that EOF drives the migration of the nonwetting phase (oil) through viscous momentum transfer
3		Jia^17^ et al	the unique advantage of EOF in overcoming low permeability to initiate pressure and establish effective flow was emphasized, as it does not rely on pressure differential drive. It was also noted that electric field drive may accelerate fluid migration in dominant pathways, leading to premature water breakthrough and reduced macro-scale ripple efficiency
4		Kokal^34^ et al	they observed that asphaltenes display electrical properties that depend on the pH, ionic strength, and salinity of the electrolyte
5		Hosseini^33^ et al	the chemical structure and complexity of bituminous materials influence their aggregation rate in the presence of an electric field
6		Azari^35^	experiments determined the charge of asphaltic colloidal particles, revealing that the EP mobility decreases with increasing particle size
7		Hashmi & Firoozabadi^22^	the net charge of asphaltic aggregates can be either positive or negative, depending on the nature of the surrounding solvent, pH value, and the presence of stabilizing gum molecules, making their behavior highly complex
8		Wuzhang^78^ et al	as the concentration of surfactants increases, the electrophoretic mobility of oil droplets typically increases (absolute value rises) until the CMC is reached
9		Ghosh & Shalabi^70^	the study investigated the effect of direct current direction on sandstone permeability and recovery rate, finding that a forward electric field (aligned with water flow direction) yielded better results, likely due to promoting clay decomposition/transportation
10		Ikpeka et al.^13^	the system reviews the effectiveness of EK-EOR, indicating that it is more effective in tight reservoirs with permeability ranging from 0.5 to 1.5 mD
11		Qi;^108^ Salipante & Vlahovska^109^	the electrohydrodynamics of droplets and jets in microsystems are discussed, indicating that the net migration direction of oil droplets ultimately results from the complex interaction of multiple forces
12	electrical heating	Yuan^38^ et al	the system reviews Joule heating in energy materials, indicating that both electrical conductivity and electric field strength jointly govern the heat generation rate
13		Lashgari^36^ et al	Joule heating was investigated through simulation, demonstrating that the heat generation rate is proportional to the square of the current density and the resistivity. The effects of mineralization degree, water saturation, and temperature on resistivity and heating efficiency were analyzed
		Kwak^43^ et al	they experimentally demonstrated localized dielectric loss heating in microscale devices, attributing the phenomenon to polarization lag and molecular relaxation
14		Yadali Jamaloei^44^	comprehensive studies indicate that RF heating can increase near-wellbore temperatures by > 120 °C and boost production capacity by approximately 30%
15		Fanchi^45^	when σ/ε′ is low (low conductivity, high dielectric), energy is primarily converted to heat via dielectric losses, with temperature increases concentrated in dielectric material zones; when σ/ε′ is large (high conductivity, low dielectric), the proportion of Joule heating increases, but uneven current distribution can easily cause localized overheating
16		Sherwali^46^ et al	electromagnetic induction heating was simulated for the athabasca oil sands, predicting a temperature increase of approximately 100 °C and a 20–25% improvement in recovery rates
17		Alomair^47^ et al	laboratory comparisons of resistance heating, electromagnetic induction, and microwave heating revealed that microwave heating offers superior performance and energy efficiency under specific conditions
18		Hiebert^82^ et al	they found through simulation studies that electrical heating can also be used as a preheat for steam flooding or other thermal recovery methods to improve initial injectivity
19		Sahni^83^ et al	they clarified the mechanism of action of low-frequency resistive heating and high-frequency electromagnetic heating. Low-frequency heating relies on Joule heat generated by the conductivity of the formation water and is achieved by current conduction between the electrodes. High-frequency heating mainly utilizes the rapid rotation of dipoles of polar molecules in the reservoir fluid, which try to align with the rapidly oscillating electric field, causing molecular friction and generating heat
20		Yu^84^ et al	they carried out a numerical simulation study of electromagnetic heating, establishing a multiphysics coupled mathematical model. They considered the effect of temperature on heavy oil viscosity, the start-up pressure gradient of non-Darcy flow, and the dielectric properties of reservoirs. The results showed that this technology can effectively reduce viscosity and the start-up pressure gradient, and improve heavy oil fluidity, which can significantly increase heavy oil production
21		Wan^85^	they found that air-assisted steam injection was superior to electrically assisted air injection. The key difference is that the synergistic effect of steam heating and air oxidation creates a larger high-temperature combustion zone, whereas electric heating is limited by the decrease in electrical resistance due to high water saturation, and is therefore more suitable for small-scale wellbore decommissioning
22	electrochemistry	Amrouche^40^ et al	research on the application of DC heating combined with nanofluids in oil-saturated carbonate reservoirs proposes mechanisms for electrowetting and nanofluid adsorption
23		Zhang^52^ et al	experimental research on the effect of DC voltage on the wettability of dense sandstone reveals the roles of chemical oxidation and increased surface roughness
24		Karna^53^ et al	they investigated the mechanism of contact angle change under a DC electric field by molecular simulation. They found that the change in interfacial hydrogen bonding structure and the increase in viscous force in the interfacial layer lead to a decrease in contact angle
25		Tao&Tang^62^	they investigated the mechanism of viscosity reduction in paraffin-based crude oil treated with electric fields, proposing that wax particles undergo polarization and aggregate to form short chains
26		Xu^50^ et al./Yim^51^ et al	it has been discovered that electric fields can alter the surface potential of rock minerals, disrupting the electrostatic bonds that adsorb polar crude oil components—such as asphaltenes—to the rock surface, thereby causing their desorption
27		Gallo^39^ et al	it is noted that Joule heating correlates with current density, electric field strength, and dielectric conductivity. Temperature changes alter EOF rates and ion/chemical reaction rates, thereby influencing overall electrokinetic transport
28	electric field-assisted composite technology	Ansari^71^ et al	research on electric field-assisted low-concentration acidizing EOR has demonstrated that it can enhance displacement efficiency and permeability while increasing acidization depth
29		Liu^75^	it was found that the electric field induced the oil droplets to move in a fixed direction and agglomerate, and the cationic surfactant CTAB led to faster deformation/agglomeration due to the attraction with the asphaltene at low electric field strength
30		Hamid^97^ et al./Adil^98^ et al./Gharibshahi^99^ et al./Hasani^100^ et al./Afrooz^91^ et al	systematically investigate the synergistic EOR effects of electromagnetic fields and magnetic nanofluids, revealing multiple synergistic mechanisms
31		Wang^102^ et al	a novel method combining RF heating with conductive medium injection into cracks is proposed to enhance the heating range and efficiency
32		Chen^76^ et al./Li^77^ et al	surfactant systems can change the viscosity by altering the interparticle forces through interfacial polarization under an electric field, thus improving the mobility ratio
33		Soliman^107^ et al./Maksyutin^105^ et al./Maddirala^106^ et al./Ageev^104^ et al	PPT can significantly increase the porosity and porosity connectivity of sandstone, with the effect becoming more pronounced with an increased number of pulses
34		Rahman^93^ et al	they investigated the effects of SiO_2_, Al_2_O_3_, ZnO NPs, EK, and mixing techniques on EOR for carbonate reservoirs, and the SiO2NPs synergistic with EK enhanced the recovery rate by 15.2–17.6%. The mechanisms include wettability change, interfacial tension reduction and directional seepage
35		Shan^94^ et al	they analyzed the effects of DC and temperature on the adsorption kinetics of perfluorooctanoic acid (PFOA) on activated carbon by modeling and experiments, and found that DC field and low temperature increased PFOA adsorption by 38%
36		Considine^101^ et al	RF Heating and Supercritical Fluid (RF-CF) Synergistic Technology
37		Zhang^95^ et al	the experiment compared the effectiveness of SAGD, EH-SAGD, and EHES-SAGD in extra-heavy oil, finding that EHES-SAGD delivered the best results while consuming less energy
38		Shah^92^	the electric field may affect the transport pattern of NPs, which can block high-permeability channels or pore throats and divert the fluid flow to unswept areas

## 5 Field Application Status and Economic Analysis

### 5.1 Field Application Status

EEOR technology has transitioned from laboratory research to field applications through a series of pilot tests and demonstrations, among which DC-EEOR technology is more prominent.

(1) The field trial in the Santa Maria Basin, California (USA), lasted 6 years of data processing and experimental testing.^15^ The test was conducted in a 100 ft-thick unconsolidated sandstone reservoir at a depth of about 2800 ft from the surface, where conventional production enhancement techniques of steam flooding and steam throughput were unsuccessful in recovering the reservoir. Comparison of the test results for EEOR: the production without production enhancement measures was 5 bbl/d, with a crude oil gravity of 8.1 API, water cut of 45%, and gas production of 1750–2000 SCFPD, 1197 BTU/cu ft of gas, and 2290 ppm of H_2_S. After implementation of EEOR, the production rate increased to 50 bbl/d (10.7° API) with only 12% water, 3800 SCFPD, 1730 BTU/cu ft, and 2–40 ppm of H_2_S.

The EEOR technology reduced the viscosity of the crude oil and observed a reduction in water cut from 45% to 12%, suggesting that the water may have been vaporized by heating to form steam. In addition, the EEOR technology increased natural gas production by nearly two times and increased the internal energy value of the produced gas by a factor of 1.5, while virtually eliminating the production of H_2_S. The observed increase in H_2_ content through EEOR technology indicates that electrochemical reactions generate hydrogen radicals, which facilitate the “cold cracking” of heavy oil molecules. This suggests that electrochemical or electrocatalytic effects on the mineral surface are at play, extending beyond mere Joule heating. This electrochemical activity is a significant factor. The productivity of EEOR technology is approximately 185 times higher than steam throughput and 613 to 1083 times higher than steam flooding. The reservoir is located at a depth of about 2800 ft, which is beyond the range of 2000 to 2500 ft. EEOR is still effective at depths of more than 10,000 ft, which is far beyond the practical limits of steam flooding. However, subsequent field tests were discontinued because operators could not solve the sand plugging issues caused by sand production.^15^

Table 5 presents the key data that supports the above description. Furthermore, the operating costs of the EEOR technology in the Santa Maria Basin were compared with those of the steam flooding technology in the San Joaquin River Valley (Table 5). The major design and construction costs are comparable between the two, but the cost of steam flooding is a one-time investment, while EEOR is a later incremental investment to maintain project implementation. Not only is the annual energy value of EEOR much lower than that of steam flooding, but the costs of nonenergy-related maintenance costs are also lower than those of steam flooding. In fact, EEOR technology equipment requires virtually no maintenance and can be operated remotely. This means that the average lifetime of EEOR equipment is longer than that of steam flooding.

**Table 5:** Consolidated Techno-Economic Data for the Santa Maria Basin Field Trial (Data Sourced from [15])^a^

parameter	baseline	after EEOR	notes
BOPD	5	50	∼10× increase
water cut	45%	12%	significant reduction
oil API gravity	8.1°	10.7°	in situ upgrading
H_2_S content (ppm)	2290	2–40	virtually eliminated
energy efficiency (BTU/bbl)	–	7014	compared to 1.3–7.6 M for steam methods. (Prior unsuccessful EOR technologies, for this field had included steam flood and cyclic (Huff and Puff) steam injection)
non-energy O&M Costs (USD/bbl)	–	0.45	compared to 2.23 for steam flood in the San Joaquin river valley
capital, design, & installation costs (USD)	–	3,125,300	comparable to steam flood (3,400,000 USD) in the San Joaquin River Valley

a Note: This table was created by the authors to consolidate performance and techno-economic data. Data for this table were adapted from refs 59 and 60.

This case is a classic demonstration of multiple mechanisms acting in concert: the significant viscosity reduction and water vaporization are direct evidence of the Joule heating effect described in Section 2.2, while the increase in API gravity and the generation of hydrogen radicals strongly support the occurrence of in situ electrochemical upgrading and redox reactions as detailed in Section 2.3.

(2) Two horizontal heavy oil wells in Alberta, Canada, were studied:^110^ one in a mature depleted reservoir and the other in an undeveloped reservoir. The mature depleted reservoir had a depth of ∼590–610 m TVD, permeability 500 mD, and cold recovery production ∼10 bbl/d (below the standard threshold of 20 bbl/d). The MI heater cables form a single-phase (DC) system. After heating, the oil viscosity was reduced from 10,000 cP, and production was enhanced to 40–50 bbl/d with an energy ratio of 6.8–7.0 (Figure 13). The heated wells increased production by about 8750 bbl over two years.

**Figure 13:**
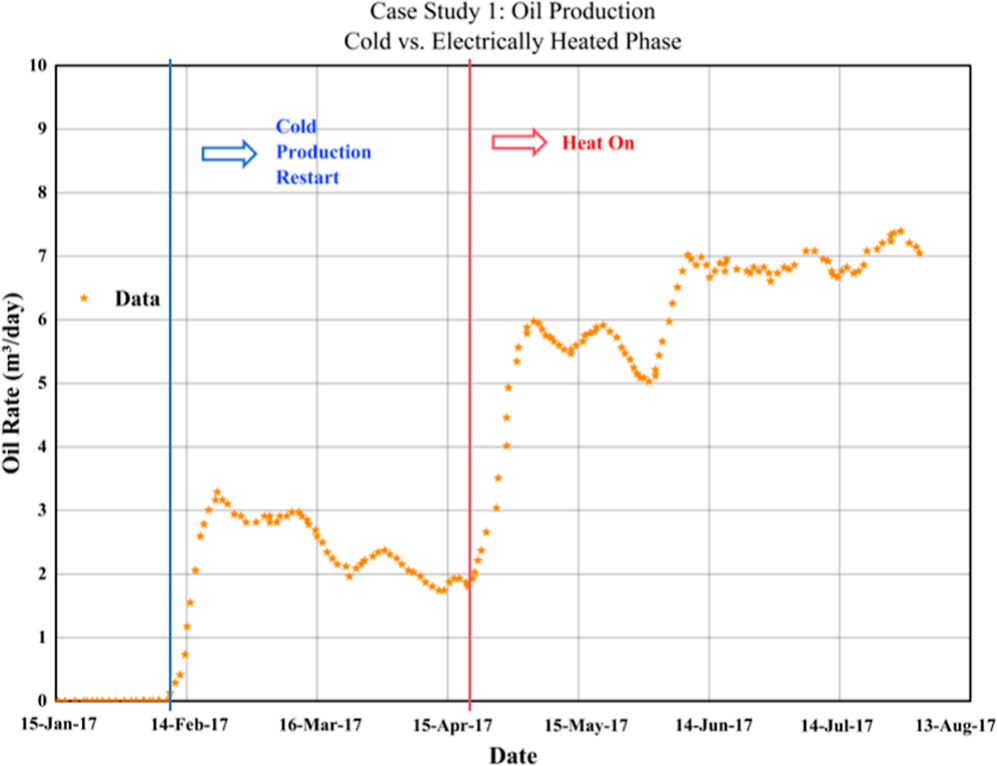
Oil rate during the “baseline” and heated production phases (Data sourced from^110^).

The undeveloped reservoir has a reservoir depth of ∼410 m TVD, permeability of 1350–2500 mD, and initial oil viscosity of 50,000–200,000 cP. After heating, the oil viscosity is significantly reduced, the production is enhanced from inefficient to ∼220 bbl/d, and the excessive output of dissolved gas is suppressed, and the production is positively correlated with the temperature (Figures 14 and 15).

**Figure 14:**
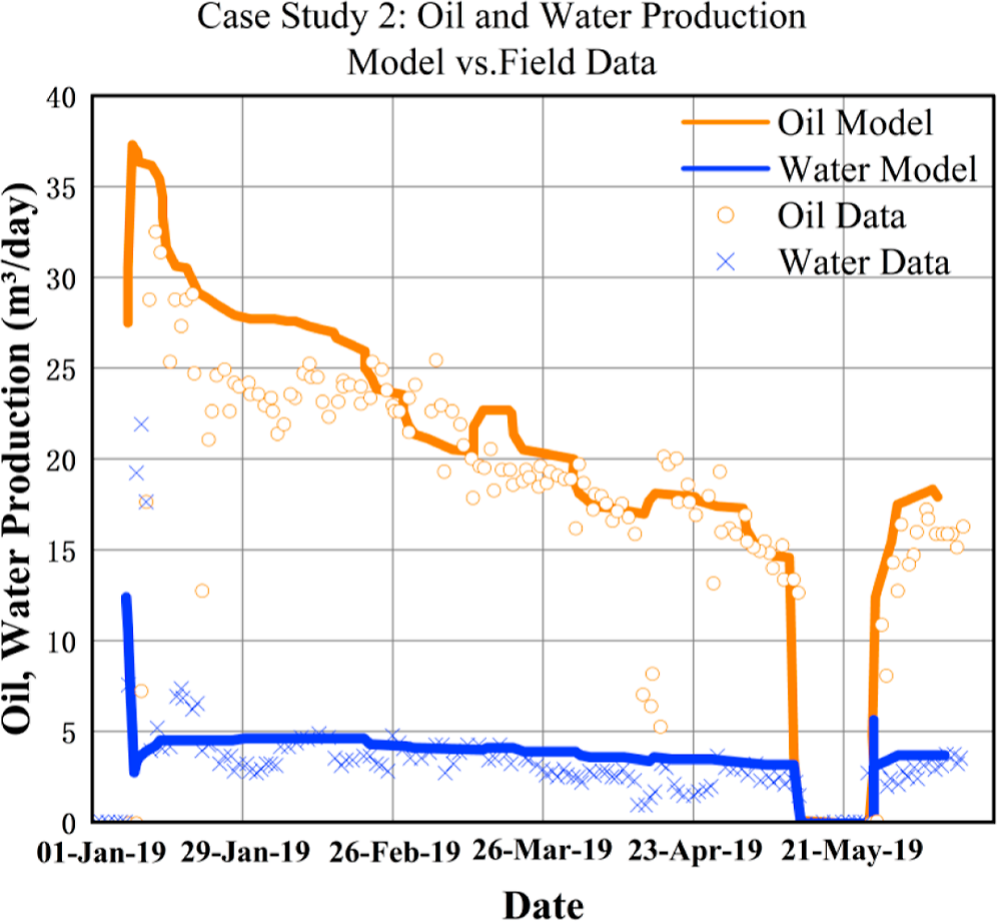
Production rates from the heated production well (Data sourced from^110^).

**Figure 15:**
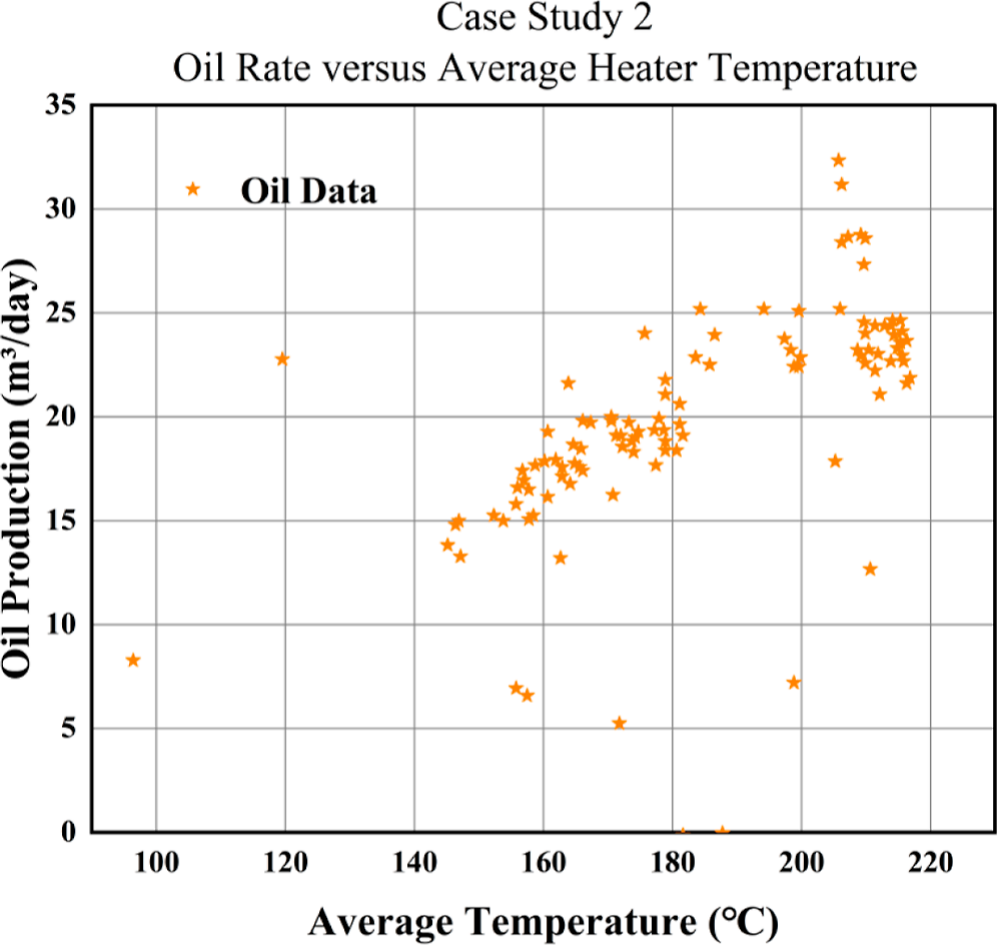
Correlation between average operating temperature and oil rate (Data sourced from^110^).

These two wells perfectly illustrate the dominant role of the electrothermal effect (Section 2.2) in heavy oil reservoirs, where the primary barrier to production is high viscosity; the direct correlation between increased production and heating confirms that thermal conduction from the downhole heater is the key recovery mechanism.

(3) The Rio Panon reservoir in Brazil is a high-permeability loose sandstone with a crude oil viscosity of 2500 cP, but with a small formation thickness of ∼28 ft (8.5 m), making it unsuitable for thermal recovery. It has a permeability of 4000 mD, a porosity of 27%, and an initial water saturation of 38%. With 60 days of primary production and no heat input, the overall average production rate was 1.2 bbl/d. This work is confined to the resistive heating process, which is the major mechanism when DC or low-frequency AC (up to 300 cycles/sec [300 Hz]) is used. After electrical heating, the 70 day production jumped from 1.2 bbl/d to >10 bbl/d, indicating a significant improvement in well production after heating.^111^

This field test provides a clear example of Joule heating’s effectiveness (Section 2.2), successfully overcoming the limitations of conventional thermal methods in thin reservoirs and demonstrating that in situ electrical heating can be a viable alternative for viscosity reduction.

(4) The EEOR field trials in the Eastern Alberta Plains included data analysis and laboratory experiments that lasted over two years,^15^ and then terminated when the client completed all heavy oil operations due to the shrinking national crude oil market. The test reservoir in Eastern Alberta has an 11 m thick unconsolidated sand layer at a depth of about 517 m below the surface and a 1.5 m thick shale interlayer 3 m below the top of the layer. Initial production under pumped conditions was approximately 0.21 m^3^/d (heavy oil) at 73% BS&W. The EEOR cycle test in Eastern Alberta showed an average EEOR production of 0.91 m^3^/d with BS&W reduced to 7% (Figure 16). Liquid levels in neighboring observation wells also rose about 60 m when the EEOR system was activated. Crude oil production efficiency was about 157 MJ/m^3^. In this shallow heavy oil field EEOR production efficiency is comparable to the effect of steam flooding.

**Figure 16:**
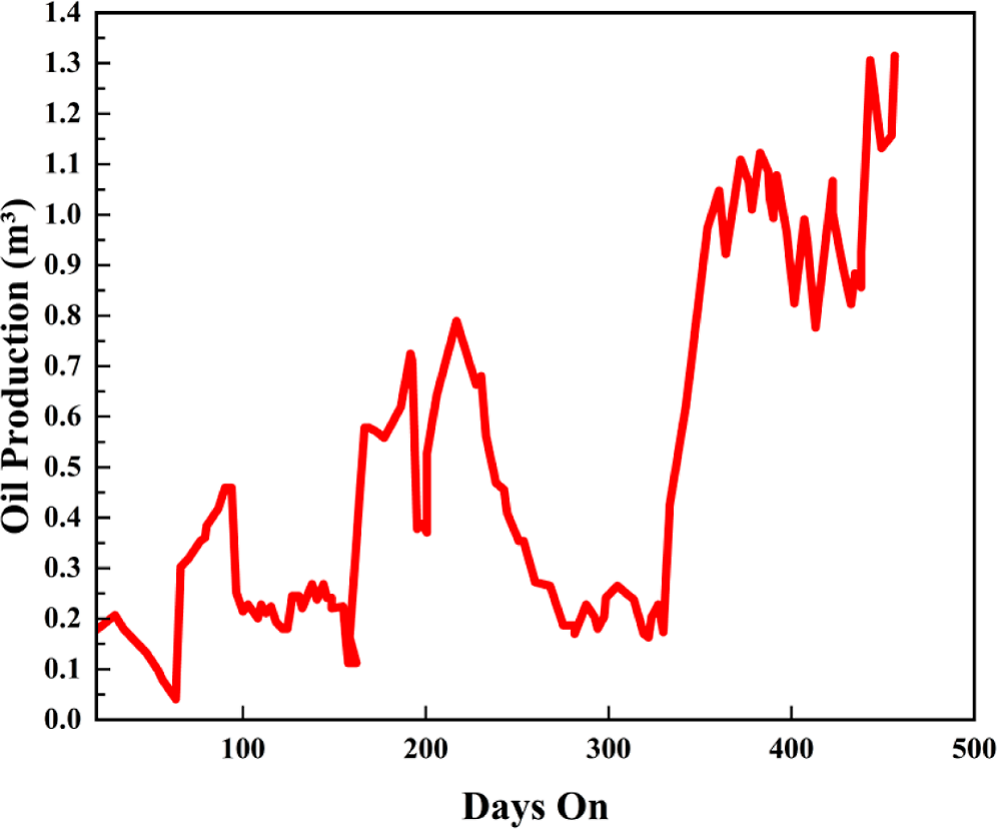
Eastern Alberta plains EEOR field demonstration (Data sourced from [^15^]).

This case highlights a synergy between mechanisms; while the application in a heavy oil field implies the contribution of Joule heating (Section 2.2), the significant rise in liquid levels in observation wells is a strong indicator of a macroscopic fluid drive, which is the signature of electroosmotic flow (EOF) as described in Section 2.1.

(5) The Golfo San Jorge Basin in Santa Cruz Province, Argentina, was investigated for the effectiveness of electric field technology in consolidated sandstone reservoirs using DCEOR field tests.^111^ The Golfo San Jorge Basin field demonstration reservoir was a 20m thick consolidated, braided stream sand with a burial depth of about 820 m. This DCEOR demonstration well was an original field without any modifications. Baseline production was approximately 11.46 bbl/d of 15.17° API heavy oil with a BS&W of 96%. The average DCEOR production was 69.79 bbl/d with BS&W down to 61%, representing a 5.09 times increase in production compared to the baseline value of 11.46 bbl/d. The DCEOR wells were converted to production wells with a BS&W of 96%. The effectiveness of EEOR technology in highly water-bearing heavy sandstone reservoirs was verified (Table 6).

**Table 6:** Golfo San Jorge Basin (Santa Cruz, Argentina) DCEOR Field Demonstration to Baseline Field Performance (Data Sourced from^111^)

item	baseline production	DCEOR production
production rate (BOPD)	11.46	69.79
water cut (%)	96	61
oil API gravity	15.17°	18.42°
viscosity (cps @ 50 °C)	2854	640

The observed results, including a drastic reduction in viscosity and a significant increase in API gravity, point to the combined action of Joule heating (Section 2.2) and electrochemical upgrading (Section 2.3), demonstrating the technology’s ability to not only mobilize but also improve the quality of the heavy oil in situ.

(6) The core principle of the ET-DSP technology in the Athabasca oil sands in Alberta, Canada, is that low-frequency electric current heats the formation, and the current carries the heat to the oil formation, creating a dual heating of “electrothermal + convection”, i.e., combining convective heat transfer and electro-osmotic effects. The conducting path for electrical current is through the continuous connate water that envelops the nonconductive sand particles. Electrical energy is converted to heat along these pathways and the heat is transferred to the oil and sand particles by conduction. The large surface area between the water film and the sand particles facilitates the rapid transfer of heat. It reduces the viscosity of the bitumen and drives it to the production wells. In the production well, the oil sands reservoir is located under a 51 m deep overburden with a net thickness of about 30 m, 30% porosity, 80% oil saturation, and raw bitumen reserves of about 10,000 barrels. The production cycle of the ET-DSP is divided into three phases: Preheating phase (about 30 days): heating the oil sands by electrodes. Heating and production phase (180 days): production while heating. Residual heat production phase: the electrodes are turned off and production continues using residual heat. The production results are shown in Table 7, where the recovery rate actually reached 77%, slightly higher than the predicted 75%. The energy consumption per barrel of bitumen was 62 kWh, 23% lower than the predicted 80 kWh, with an equivalent SOR of 0.49, showing high energy efficiency. Water use was 0.90, lower than the predicted 1.0, and peak production rate reached 18.5 bbl/d per well, higher than the predicted 15.8 bbl/d per well.^112^

**Table 7:** Model-Proof of Concept Comparison (Data Sourced from^112^)

data	model	pilot	note
recovery factor (%)	75	77	exceeded
energy oil ratio (kWh/bbl)	80	62	better than predicted (−23%)
equivalent SOR	0.56	0.49	better than predicted
net energy ratio (kJ/kJ)	26.37	30.02	better than predicted
water–oil ratio (m^3^/m^3^)	1.0	0.9	better than predicted
peak production rate (bbl/D per X-Well)	15.8	18.5	better than predicted

This project explicitly identifies the process as the Joule heating effect generated in the raw water (Section 2.2) and acknowledges the contribution of the electroosmotic effect (Section 2.1) to convective heat transfer and fluid-driven mechanisms.

(7) Regarding the Asphalt Ridge Pilot Test in Utah, USA, one of the earliest and most representative field trials of electromagnetic heating (specifically RF) for in situ bitumen recovery was conducted at the Asphalt Ridge tar sand deposit in Vernal, Utah, during the early 1980s. The objective of the pilot was to evaluate the feasibility of RF electromagnetic heating for low-water-content bitumen sands, a challenging reservoir type for conventional steam-based thermal recovery.

A triplate electrode configuration was installed to confine the electromagnetic field and improve energy coupling efficiency. RF power in the range of 40–75 kW was applied at frequencies between 2.2875 and 13.56 MHz, heating a 25 m^3^ oil sand block. Within approximately 3 weeks of operation, the formation temperature increased to 120–200 °C, and 35% of the original bitumen in place was recovered.

The heating process reduced the viscosity of bitumen from over 10^6^ cP to around 100 cP, enabling gravity drainage and gas drive. Experimental observations also revealed autogenous steam and light hydrocarbon generation, which contributed to additional production drive. The energy efficiency ratio (produced energy vs input energy) ranged between 3 and 12, indicating a favorable energy balance compared to steam injection.

This pilot test demonstrated that RF heating could effectively establish a volumetric heat front in oil sands, even at low water saturations, and provided early technical evidence for subsequent large-scale projects such as Athabasca and ESEIEH. Summary of the key results (Figure 17): RF power: 40–75 kW (2.2875–13.56 MHz); heated volume: 25 m^3^; temperature rise: 120–200 °C; recovery factor: 35% OOIP; energy ratio: 3–12; duration: ∼3 weeks.^113^

**Figure 17:**
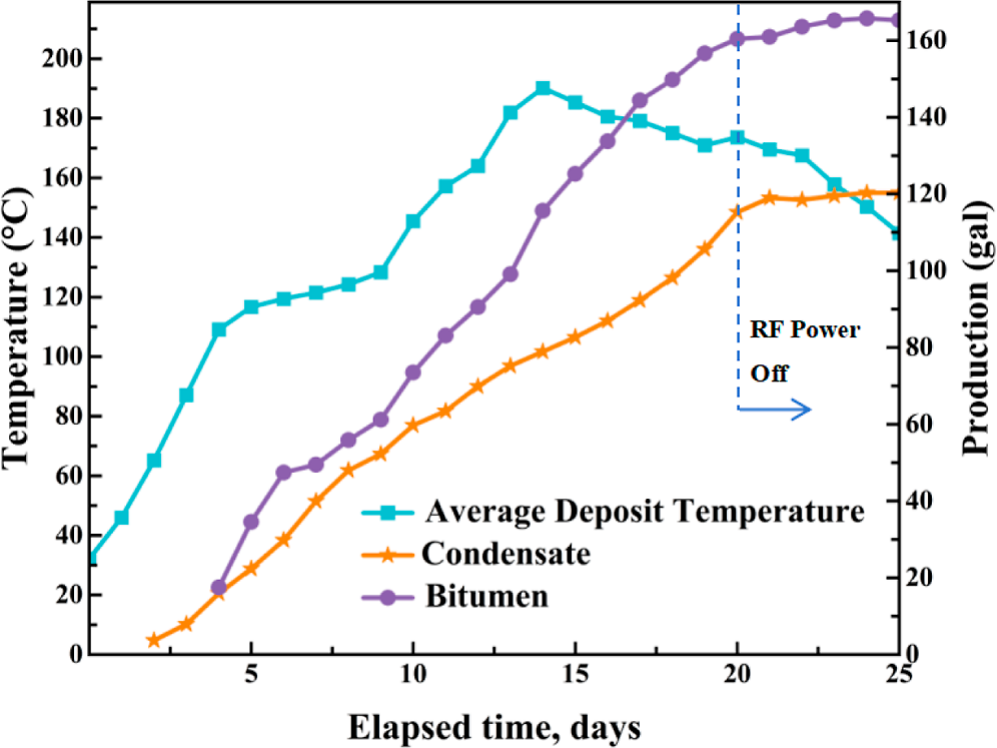
Average temperature of deposit and cumulative production of bitumen (Data sourced from^113^).

This pilot project serves as a typical demonstration of RF heating within the electrical heating (Section 2.2). Through the combined action of in situ dielectric loss heating via high-frequency electromagnetic waves and Joule heating, it elevates formation temperatures and significantly reduces asphalt viscosity. This validates the feasibility of electromagnetic heating for volumetric heating and thermal-driven processes in oil sands with low water saturation.

(8) Figure 18 presents experimental data from the field application of PPT in selected Russian oilfields. The percentages in the figure represent the improvement in BOPD achieved after applying PPT technology. After PPT treatment, BOPD was significantly higher than before treatment, with particularly pronounced effects on low-yielding wells. In most fields, water cut decreased post-treatment. The Krapivinskoe field experienced a slight increase in water cut (from 70% to 77%) following treatment, potentially related to specific geological or engineering conditions at that well. Overall, this technology demonstrates strong potential for enhancing oil recovery and reducing water cut.

**Figure 18:**
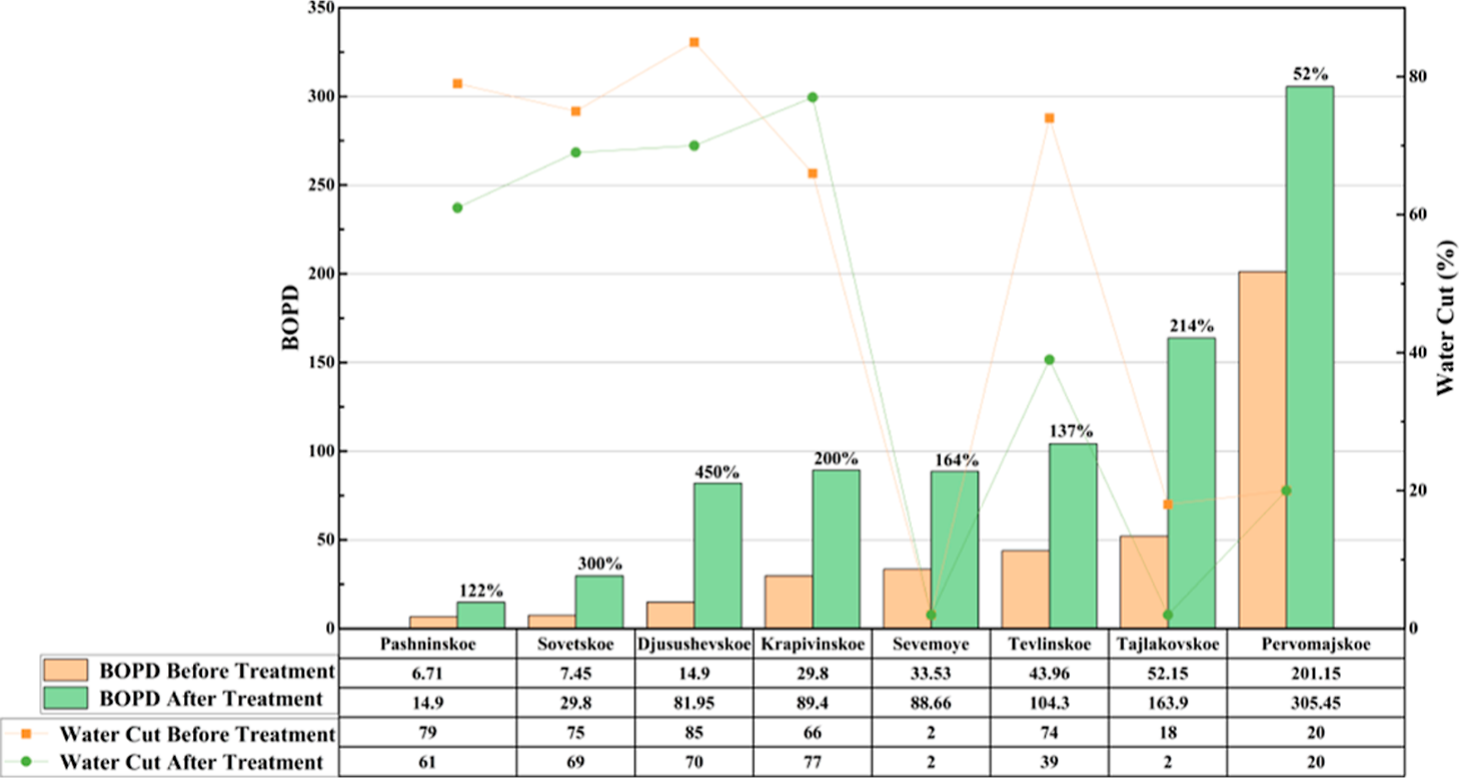
Oil production and water cut before and after of PPT treatment on production well of few oil fields of Russia (Data sourced from Novas Energy, 2015)[Fn fn1].

These data validate the field effectiveness of PPT (Section 4.2.3), a technology that utilizes shock waves generated by high-voltage pulse discharges to enhance permeability around the wellbore. Simultaneously, it may reduce fluid viscosity by disrupting the thixotropic structure of bitumen, thereby increasing production and lowering water cut.

(9) Kuwait Oil Company (KOC) successfully applied PPT for the first time to enhance production at its RA-000A well, located in the Lower Burgan (LB) reservoir northeast of the Burgan oilfield. The primary objective of applying PPT was to boost production from the extremely low-permeability SID2 zone, with the expectation of positively impacting the adjacent RA-000B well 400 m away. Prior to treatment, the well was perforated in three zones, with a last reported production rate of 196 barrels per day (BOPD) and a water cut as high as 90%. Following the decision to plug the high-water-cut COAL2_UCH layer and perforate the low-permeability SID3 layer, models projected the well’s production potential at 800–1000 barrels liquid per day (BLPD), with an expected production index (PI) of 1.0–1.5 barrels per day per psi. One month after PPT operations, actual test results showed the well achieved a stable production of 1279 BLPD, with oil production at 363 BOPD. The actual PI rose to 2.25 barrels per day per psi—nearly double the pretreatment PI and significantly higher than model projections. This indicates PPT delivered a net oil gain of 167 BOPD, representing an 85% increase over initial production. Although liquid volume and PI exceeded expectations, the actual water cut reached 71%, higher than the model projection. Furthermore, immediately following the PPT operation, the pump inlet pressure in the adjacent well RA-000B increased by 81 psi (from 1441 to 1525 psi), while the water cut decreased from 48% to 38%, demonstrating the potential scope of this technology’s impact.^114^ This case study provides further field evidence for the PPT method (Section 4.2.3), demonstrating that high-energy electrical pulses successfully modified low-permeability formations. The generated mechanical shock waves created new fractures and enhanced formation permeability, significantly improving the PI and even inducing pressure response in adjacent wells.

Table 8 is introduced here to synthesize and compare the techno-economic performance of various EEOR field trials against conventional EOR methods. This table collocates key performance indicators from disparate sources into a single, structured format, allowing for a direct, quantitative comparison of energy efficiency and cost. This synthesis transforms anecdotal claims about EEOR’s economic advantages into a data-driven argument, providing a clearer picture of its competitive positioning.

**Table 8:** Comparative Techno-Economic Metrics of EEOR Field Trials and Conventional EOR Methods

EOR method	field/basin	reservoir type	incremental recovery/production increase	energy efficiency metric	capital costs (USD)	operating costs (USD/bbl)
DC EEOR	Santa Maria Basin, CA	heavy oil, sandstone	5 to 50 BOPD (10× increase)	7014 BTU/bbl	3,125,300	0.45 (nonenergy O&M)
electrical heating	Rio Panon, Brazil	high-permeability, loose sandstone	1.2 to >10 BOPD (>8× increase)		–	
steam flood	San Joaquin Valley, CA	heavy oil, sandstone		4.3–7.6 M BTU/bbl	3,400,000	2.23 (non-energy O&M)
electrical heating	Alberta, Canada	heavy oil, depleted	∼10 to 40–50 BOPD (4–5× increase)	energy ratio: 6.8–7.0		
ET-DSP	Athabasca, Canada	oil sands	77% Recovery factor	62 kWh/bbl (SOR: 0.49)		
CO_2_ flooding^24^	Generic	various	5–20% of OOIP		highly variable (CO_2_ source and transport)	highly variable (CO_2_ purchase price)
RF heating	Asphalt Ridge Pilot Test, Utah, USA	tar sands	35% OOIP	3–12	ranging from 85.4 million to 120.3 million USD^a^	ranging from 5.88 to 12.68 USD/barrel

a Note: Costs are an economic projection (in 1981 USD) for a 10,000 B/D commercial facility with a 20 year life, not the actual cost of the pilot test.

Despite its demonstrated potential, the slow transition of EEOR from pilot-scale success to widespread commercial deployment is primarily hindered by a series of significant operational, engineering, and economic challenges. Table 9 summarizes its challenges and the fundamental reasons behind them.

**Table 9:** Summary of Key Operational Challenges in EEOR

challenge	primary root cause(s)
downhole hardware failure	harsh environment (corrosion, HTHP, H_2_S), material fatigue, electrical stress, current overload
high energy consumption	inefficient current pathways (bypassing oil), heat loss to surrounding formations, low formation conductivity
reservoir/wellbore integrity	electric-field-induced changes in geomechanical stress; rapid fluid/gas production destabilizing formations
unpredictable performance	complex multiphysics interactions (antagonism/synergy), significant impact of reservoir heterogeneity

Electrical heating methods (DC EOR, Electrical Heating, ET-DSP, RF Heating) consistently demonstrate superior energy efficiency and significant production enhancement when treating heavy oils such as oil sands. Compared to conventional steam flooding, electrical heating methods consume substantially less energy and, in specific cases, incur lower nonenergy operating costs. DC EOR delivered outstanding overall performance (production enhancement, energy efficiency, cost) in the Santa Maria case study. ET-DSP demonstrated exceptionally high recovery rates and favorable energy efficiency in the Athabasca case. RF heating offers high energy efficiency, but based on preliminary estimates, its costs for large-scale deployment may be relatively high. Overall, for specific reservoir types-particularly heavy oils and deep/thin reservoirs—electrical heating methods appear to offer greater energy efficiency and cost advantages over steam flooding, while delivering substantial production increases or high recovery rates. Additionally, although EEOR technology has been researched for decades, well-documented field applications remain limited, and most of the field data are derived from pilot tests. Among the reported EEOR field applications, heavy oil reservoirs are by far the most applied, with thermal effects and in situ electrochemical reforming playing a dominant role in heavy oil recovery. Moreover, other fields lack publicly available detailed data to provide empirical and comprehensive assessment of field operations and quantitative results. The translation of this technology to field applications has been relatively slow, which may be affected by factors such as economic considerations, operational complexity, or inconsistency of results under different reservoir conditions. While studies claim that EEOR is less costly than other EOR technologies and has good potential for economic benefits, the cost of electricity, investment in equipment, and the uncertainty of achieving the desired recovery rate under specific reservoir conditions remain challenges. Corrosion of electrodes and wellbore materials is also a serious issue in high salinity, high temperature and electrochemically active environments. For instance, some field applications report failure rates as high as 75% for electrodes, primarily due to cable breakage from overloading.^112^ Therefore, a number of factors need to be considered when designing field tests.

### 5.2 Economic Analysis

#### 5.2.1 Economic Framework and Cost Breakdown

A comprehensive economic assessment of EEOR projects involves breaking down the cost structure into two core components: capital expenditure (CapEx) and operating expenditure (OpEx). CapEx primarily consists of:

(1) Surface facilities: this is the main capital expenditure item, including power generation equipment, high-voltage transformers, switchgear, motor control centers, and complex instrumentation and control systems. Additionally, it includes engineering costs such as equipment foundations and construction.
(2) Underground infrastructure: this includes costs associated with drilling new wells or performing well maintenance on existing wells to install electrodes or heaters.
(3) Indirect and auxiliary costs: these encompass all expenses from feasibility studies, project design, engineering services, construction management, to environmental impact assessments and related costs.

OpEx primarily includes:

(1) Energy consumption (electricity): this is the most significant component of operational costs in EEOR projects. Total electricity costs directly depend on the system’s rated power (kW), operating time, and local industrial electricity rates (USD/kWh).^115^ Fluctuations in electricity prices are a major risk factor for project economics.
(2) Maintenance and repair costs: regular maintenance of surface electrical equipment and downhole components is essential. Downhole equipment operates in harsh environments characterized by high temperatures, high pressures, and corrosive fluids, posing a high risk of failure.^116^ The costs associated with well intervention operations required to repair or replace faulty underground components are high, such as nonproductive time (NPT) caused by underground tool failures, which can result in losses of up to 1 million USD per day.^116^
(3) Labor and monitoring costs: these include expenses for technicians operating and monitoring the entire system, as well as costs for the EEOR digital monitoring system.
(4) Other costs: standard oilfield lease operating expenses (LOE), including produced fluid treatment, chemical management, and disposal of saline wastewater.

EEOR’s heavy reliance on electricity creates a strategic link in its cost structure, where the economic viability of oilfield extraction projects is closely tied to the evolution of broader energy markets. Electricity is a significant OpEx,^115^ the profitability of EEOR projects is directly tied to the dynamics of the regional electricity market. This implies that oil companies considering the implementation of EEOR must transcend traditional oil and gas exploration and production thinking and fundamentally transform their traditional oil and gas production business model into an integrated energy operations model.

#### 5.2.2 Economic Comparison Benchmark Withconventional EOR

To assess the economic competitiveness of EEOR, it must be directly compared with mature conventional EOR technologies across multiple dimensions. This comparison reveals the unique advantages and limitations of EEOR in specific application scenarios.

(1) Comparison between EEOR and steam flooding: EEOR offers significant advantages in energy utilization, with the energy output/input ratio of electric heating technology exceeding 20,^117^ while steam flooding suffers from severe heat loss, with a steam-to-oil ratio (SOR) typically ranging from 5 to 7 in most field projects.^118^
(2) Comparison between EEOR and chemical flooding: this is a comparison of electricity costs versus chemical agent costs. Chemical agents are expensive, and a case study indicates that chemical agent costs alone could reach 3.12 to 7.83 USD per barrel of incremental oil production. Laboratory corefloods using field-proportioned volumes of chemical solutions injected with dead oil and reservoir rock resulted in average chemical costs of 3.12 USD per incremental barrel of oil for alkaline surfactant–polymer formulations and 18.61 USD for surfactant–polymer formulations.^119^ In contrast, thermodynamic methods in EEOR are based on more predictable physics, potentially resulting in lower technical risks.
(3) Comparison between EEOR and gas (CO_2_) flooding: CO_2_-EOR requires extensive infrastructure, including a gas source, transportation pipelines, and a circulation injection system.^120^ Injected CO_2_ can also form acidic substances that corrode equipment.^121^ EEOR infrastructure is relatively simple and avoids potential corrosion issues.

EEOR’s primary competitive advantage lies not in being universally cheaper than traditional methods, but in its role as an enabling technology. It can unlock “stagnant” reserves that have been set aside due to technical limitations. For example, for reservoirs that are too deep, too thin, fractured, or located in water-scarce regions, making conventional EOR methods (such as steam flooding) technically infeasible, EEOR offers a better solution. In these scenarios, EEOR’s competitors are not other EOR technologies but the “zero production” baseline. Therefore, its economic threshold is relatively low, making it an extremely attractive option for creating new value.

#### 5.2.3 Economic Analysis of Field Examples

Although not all EEOR field trial literature includes comprehensive economic evaluations, some detailed case studies provide strong evidence of the economic potential of EEOR technology. The following two oilfields serve as examples:

In trials conducted in the Santa Maria Basin in California, USA, EEOR technology demonstrated significant effectiveness. Economically, the production efficiency of this technology far exceeds that of traditional thermal recovery, being approximately 185 times that of steam flooding and 613 to 1083 times that of steam flooding. Compared to steam flooding, EEOR significantly reduces annual energy costs (86,200 USD vs 620,800 USD) and nonenergy operational costs (0.45 USD/barrel vs 2.23 USD/barrel), while also offering a longer expected equipment lifespan. However, the trial was halted due to sand blockage issues caused by sand production.^15^

In trials conducted on two horizontal heavy oil wells in Alberta, Canada, electric heating technology achieved success in reservoirs at different development stages. For a mature depleted reservoir, electric heating increased production from approximately 10 barrels per day to 40–50 barrels per day, with an energy input–output ratio of 6.8–7.0.^110^ Similarly, downhole resistive heating in this reservoir achieved oil production rates 4 to 6 times higher than the unheated reference well, with an energy ratio exceeding 20.0, demonstrating extremely high energy efficiency.^117^

The ET-DSP electric heating pilot test conducted in the Athabasca oil sands in Canada outperformed or met all model predictions. Energy efficiency was outstanding, with energy consumption per barrel of bitumen as low as 62 kWh and a steam-oil ratio (SOR) as low as 0.49. Although the trial was successful, it also revealed operational challenges such as electrode cable overload.^112^

Although some reports have demonstrated its potential economic viability, actual commercial-scale deployment cases remain scarce. This phenomenon suggests that while EEOR technology is technically robust, its successful transition from “pilot field” to “large-scale field” may face significant unresolved economic challenges. First, the cost of deploying and maintaining reliable power infrastructure on a large-scale oilfield may be extremely high; second, the long-term reliability of downhole electrical components remains uncertain; and third, the project is highly dependent on fluctuations in power market prices. Cost control for EEOR can be achieved through the following methods:

(1) Material and equipment improvements: more durable, high-temperature-resistant, and cost-effective downhole heating cables and electrodes need to be developed. Particularly in terms of conductor, insulator, and sheath materials, these improvements are critical for enhancing the reliability and lifespan of downhole systems.
(2) Digitalization and automation: integrating AI into EEOR operations. By optimizing heating cycles through smart algorithms and conducting predictive maintenance on equipment health, energy efficiency can be maximized, unplanned downtime minimized, and labor supervision costs reduced.
(3) Hybrid application methods: combining EEOR with other EOR technologies may yield synergistic effects. For example, preheating reservoirs with electric heating before implementing steam or solvent flooding (e.g., electric preheating-assisted SAGD) can improve initial startup performance, shorten time to effectiveness, and optimize overall energy consumption.

## 6 Conclusions and Suggestions

### 6.1 Main Findings

After examining the persistent gap between the potential of EEOR laboratories and their limited field deployment, this paper synthesizes its analysis into four key findings. These conclusions offer a more refined perspective on the technology’s current state by highlighting its fundamental mechanism conflicts and engineering bottlenecks:

1. Gap between field application and scalability: (1) limited application: despite decades of research and extensive laboratory validation of EEOR technologies, documented cases of true field-scale implementation remain extremely scarce. (2) Data deficit: most field data originates from pilot tests, particularly in reservoir types beyond heavy oil. There is a lack of publicly available detailed data to empirically validate and comprehensively evaluate field operations and quantitative outcomes. (3) Slow commercialization: the transition from laboratory potential to field application has been relatively slow, potentially influenced by economic viability, operational complexity, and inconsistent performance across varying reservoir conditions.
2. Major operational and equipment challenges: (1) low equipment reliability: a core challenge lies in the reliability of downhole tools, such as downstream electrodes and cable systems. Corrosion poses a severe issue in harsh environments characterized by high salinity, elevated temperatures, and electrochemically active conditions. (2) High failure rates: some field applications report electrode failure rates as high as 75%, primarily due to cable overload breakage. (3) High maintenance costs: the high risk of downhole equipment failure makes well intervention operations required to repair or replace faulty subsurface components-extremely costly. (4) Geomechanical challenges: field deployment is hindered by operational challenges related to material reliability and geomechanical stability. For instance, trials in the Santa Maria Basin were halted due to unresolved sand production and subsequent wellbore blockage issues. (5) Limitations of PPT technology: key constraints of PPT include a limited production enhancement radius and logistical challenges associated with cable delivery systems.
3. Fundamental mechanism antagonism and research gaps: (1) antagonistic core mechanisms and model deficiencies: a fundamental characteristic of EEOR systems is the inherent antagonism between electrothermal and electromechanical mechanisms, as well as between EOF and EP. This conflict is primarily governed by reservoir brine salinity, where conditions favorable to one mechanism typically hinder the other. Consequently, a core research gap currently lies in the absence of coupled multiphysics quantitative models. (2) Existing models struggle to integrate factors such as droplet size, surface charge, fluid viscosity, pore throat geometry, and nonuniform electric field distribution to accurately predict net effects (e.g., under the combined influence of EOF and EP). (3) Unclear mechanistic contribution: although laboratory studies consistently demonstrate that electrowetting can significantly alter wettability, its application at field scale is complex. Compared to mechanisms affecting macroscopic fluids like EOF or Joule heating, its contribution to the overall oil recovery process may be relatively minor.
4. Economic and cost challenges: (1) uncertainty: while some studies claim EEOR is cost-effective, challenges remain regarding electricity costs, equipment investment, and the uncertainty of achieving target recovery rates under specific reservoir conditions. (2) High CapEx: Primary capital expenditures are incurred in surface facilities, including power generation equipment, high-voltage transformers, switchgear, and complex control systems. (3) High OpEx Risk: energy consumption (primarily electricity) constitutes the most significant component of operating costs. Fluctuations in electricity prices represent a major risk factor for project economics.

### 6.2 Suggestions

To bridge the gap between proven technological potential and current field-scale reliability, the future of EEOR research must pivot from simply demonstrating that the mechanisms work to solving the specific engineering, scientific, and economic problems that prevent the technology from being reliable and profitable at scale. The following targeted roadmap for future research is proposed.

1. Addressing core engineering bottlenecks: Develop cost-effective, reliable downhole electrode and cable systems capable of withstanding harsh reservoir environments to address primary failure modes observed in field testing and mitigate significant economic losses resulting from equipment failure.
2. Advancing fundamental science and modeling: Quantify antagonistic and synergistic effects between mechanisms through robust coupled numerical models, and elucidate precise electrochemical pathways for in situ petroleum modification.
3. Advancing research on synergistic composite technologies: Explore synergistic effects between EOR and other EOR techniques such as low-salinity water, chemical agents, and nanofluids. Investigate their coupled applications with multiphysical fields including acoustic and magnetic fields. Integrating multiple effects can overcome the limitations of individual technologies.
4. Implementing artificial intelligence optimization control: EEOR involves the dynamic coupling of electric, flow, temperature and chemical fields, and its optimal operating parameters tend to change with the reservoir conditions and the production stage. The introduction of artificial intelligence can help manage this high complexity and uncertainty. With the help of AI technology to process real-time monitoring data, it can realize the optimal control of the EEOR process, for instance, by dynamically adjusting voltage and current to maintain the optimal balance between electrothermal and electrokinetic effects. It not only improves efficiency, but also helps to reduce operational risks and costs.
5. Enhancing technical-economic analysis and field validation: Conduct comprehensive economic modeling of hybrid systems and initiate targeted pilot studies in new reservoir types (such as tight carbonates) to validate laboratory results and broaden the application window.
6. Establishing a link with in situ hydrogen generation: Future research should explicitly explore the potential of optimizing EEOR systems to achieve the coproduction of hydrocarbons and low-carbon hydrogen. This will involve investigating electrode materials and operational parameters to maximize hydrogen yield, by designing novel catalytic electrodes to upgrade the electrically driven recovery system into an “underground electrocatalytic reactor”, which can enable in situ heavy oil hydro-upgrading while simultaneously coproducing low-carbon hydrogen, thereby positioning EEOR technology as a hybrid energy technology.

## Footnotes

1
http://www.novasenergy.ca/technology/presentations.html.
